# Characterization of Two Novel Toti-Like Viruses Co-infecting the Atlantic Blue Crab, *Callinectes sapidus*, in Its Northern Range of the United States

**DOI:** 10.3389/fmicb.2022.855750

**Published:** 2022-03-03

**Authors:** Mingli Zhao, Lan Xu, Holly Bowers, Eric J. Schott

**Affiliations:** ^1^Institute of Marine and Environmental Technology, University of Maryland, Baltimore County, MD, United States; ^2^Department of Marine Biotechnology, Institute of Marine and Environmental Technology, University of Maryland, Baltimore County, MD, United States; ^3^Moss Landing Marine Laboratory, San Jose State University, San Jose, CA, United States; ^4^Institute of Marine and Environmental Technology, University of Maryland Center for Environmental Science, Cambridge, MD, United States

**Keywords:** dsRNA virus, totivirus, crustacean, disease ecology, host habitat expansion, next generation sequencing, taxonomy, climate

## Abstract

The advancement of high throughput sequencing has greatly facilitated the exploration of viruses that infect marine hosts. For example, a number of putative virus genomes belonging to the *Totiviridae* family have been described in crustacean hosts. However, there has been no characterization of the most newly discovered putative viruses beyond description of their genomes. In this study, two novel double-stranded RNA (dsRNA) virus genomes were discovered in the Atlantic blue crab (*Callinectes sapidus*) and further investigated. Sequencing of both virus genomes revealed that they each encode RNA dependent RNA polymerase proteins (RdRps) with similarities to toti-like viruses. The viruses were tentatively named *Callinectes sapidus* toti-like virus 1 (CsTLV1) and *Callinectes sapidus* toti-like virus 2 (CsTLV2). Both genomes have typical elements required for −1 ribosomal frameshifting, which may induce the expression of an encoded ORF1–ORF2 (gag-pol) fusion protein. Phylogenetic analyses of CsTLV1 and CsTLV2 RdRp amino acid sequences suggested that they are members of two new genera in the family *Totiviridae*. The CsTLV1 and CsTLV2 genomes were detected in muscle, gill, and hepatopancreas of blue crabs by real-time reverse transcription quantitative PCR (RT-qPCR). The presence of ~40 nm totivirus-like viral particles in all three tissues was verified by transmission electron microscopy, and pathology associated with CsTLV1 and CsTLV2 infections were observed by histology. PCR assays showed the prevalence and geographic range of these viruses, to be restricted to the northeast United States sites sampled. The two virus genomes co-occurred in almost all cases, with the CsTLV2 genome being found on its own in 8.5% cases, and the CsTLV1 genome not yet found on its own. To our knowledge, this is the first report of toti-like viruses in *C. sapidus*. The information reported here provides the knowledge and tools to investigate transmission and potential pathogenicity of these viruses.

## Introduction

With the wide application of next generation sequencing (NGS), a huge number of virus genomes have been described from studies of metagenomes and viromes. How to best use the massive amount of data produced by NGS remains a fundamental challenge. For instance, an increasing number of toti-like virus sequences have been revealed by metagenomic studies, but further characterization and investigation are lacking (Shi et al., 2016). Ideally, following NGS-based discovery, attention should also be paid to characterize the biological properties of putative viruses, especially their genetics, viral morphological characteristics, geographic range, and potential impacts on hosts.

Viruses of the *Totiviridae* family have a non-segmented, double-stranded RNA (dsRNA) genome, with two open reading frames (ORFs) encoding the putative capsid protein (Cp) and the RNA dependent RNA polymerase (RdRp). Most virions in this family are isometric with no projections, and are ∼40 nm in diameter (Wickner et al., 2011). At present, five genera are officially recognized by the International Committee on Taxonomy of Viruses (ICTV), including *Totivirus*, *Victorivirus*, *Giardiavirus*, *Leishmaniavirus*, and *Trichomonasvirus* (King et al., 2011; Wickner et al., 2011). Viruses belonging to the genera *Totivirus* and *Victorivirus* mainly infect fungi, whereas those in the genera *Giardiavirus*, *Leishmaniavirus*, and *Trichomonasvirus* are present in parasitic protozoa and do not appear to cause cytopathic effects (Ghabrial and Suzuki, 2009; Goodman et al., 2011). Recently, non-ICTV recognized totivirus species have been found in arthropod hosts, such as mosquitoes, ants, flies, as well as crustaceans (Poulos et al., 2006; Wu et al., 2010; Koyama et al., 2015, 2016). Novel toti-like viruses have also been found in fish and plant hosts (Haugland et al., 2011; Abreu et al., 2015; Chen et al., 2015). Two genera were proposed recently, including Artivirus which infect arthropod and fish hosts (Zhai et al., 2010), and Insevirus which infect insect hosts (Zhang et al., 2018).

Two totiviruses have been reported to cause crustacean disease: a Cherax *Giardiavirus*-like virus (CGV) in freshwater crayfish (*Cherax quadricarinatus*) and infectious myonecrosis (IMN) virus (IMNV) in the Pacific white shrimp (*Litopenaeus vannamei*). CGV was the first totivirus identified in crustaceans and caused high morbidity and mortality in infected juvenile crayfish (Edgerton et al., 1994). IMNV is the most well studied totivirus in crustaceans, which causes IMN in the Pacific white shrimp in Brazil and Indonesia (Lightner et al., 2004; Poulos et al., 2006; Senapin et al., 2007; Naim et al., 2014). Additionally, metagenomics studies have reported totivirus-like dsRNA genome sequences in sesarmid and charybdis crab (Refseq. NC_032566.1 and NC_032462.1) but these have not had further characterization or investigation (Shi et al., 2016).

The Atlantic blue crab, *Callinectes sapidus*, is an adaptable estuarine species that functions as both predator and prey in food webs and supports important fisheries from the United States mid-Atlantic coast to southern Brazil (Millikin, 1984; NOAA, 2020). *Callinectes sapidus* has greatly expanded its geographic habitat range since the Last Glacial Maximum when the seas became warmer (Macedo et al., 2019), and has been introduced to Asia and Europe waters as an invasive species since 1901 (Millikin, 1984; Mancinelli et al., 2021). Unique within the *Callinectes* genus, *C. sapidus* has the ability to inhabit high latitudes by becoming dormant in winter. As the climate and ocean temperatures have changed, the distribution of *C. sapidus* has shifted poleward and the abundance of *C. sapidus* has increased at high latitudes, as far north as Nova Scotia, Canada and as far south as Argentina (Piers, 1920; Gosner, 1978; Johnson, 2015).

Within the mid-Atlantic coast and Gulf of Mexico, a range of viruses have been described in blue crabs, in the families *Baculoviridae*, *Herpesviridae*, *Reoviridae*, *Picornaviridae*, *Roniviridae*, *Rhabdoviridae*, and *Bunyaviridae* (Johnson, 1978, 1983, 1984; Bowers et al., 2010; Shields et al., 2015; Zhao et al., 2021a,b). With one exception, the relationship of these viruses to the blue crab range, and climate factors are unknown. *Callinectes sapidus* reovirus 1 (CsRV1), which causes disease and mortality in *C. sapidus*, is more prevalent in blue crabs at higher latitudes (Zhao et al., 2020), which illustrates that host-pathogen interactions can be strongly affected by habitat and environmental changes. Therefore, investigations of the effects of climate-related range extension and variation on host-pathogen interactions of other viruses will advance the understanding of drivers for virus epizootiology and ecology. The feasibility of such studies has been dramatically accelerated by molecular technologies of qPCR and high throughput sequencing, enabling virus discovery and tracking (Maclot et al., 2020).

Here, we report the discovery and characterization of two novel toti-like virus genomes that co-infect *C. sapidus* along the northern Atlantic coast of the United States. Transmission electron microscope (TEM) revealed all virions are ~40 nm in diameter, suggesting that either the two viruses are in similar size or that only one of the viruses produces virions. Pathology caused by the viruses was revealed by histology. Additionally, probe-based real-time reverse transcription quantitative PCR (RT-qPCR) assays were developed to screen and quantify totivirus infections in large numbers of *C. sapidus* across a climatological gradient.

## Materials and Methods

### Crab Sampling

**Figure 1:**
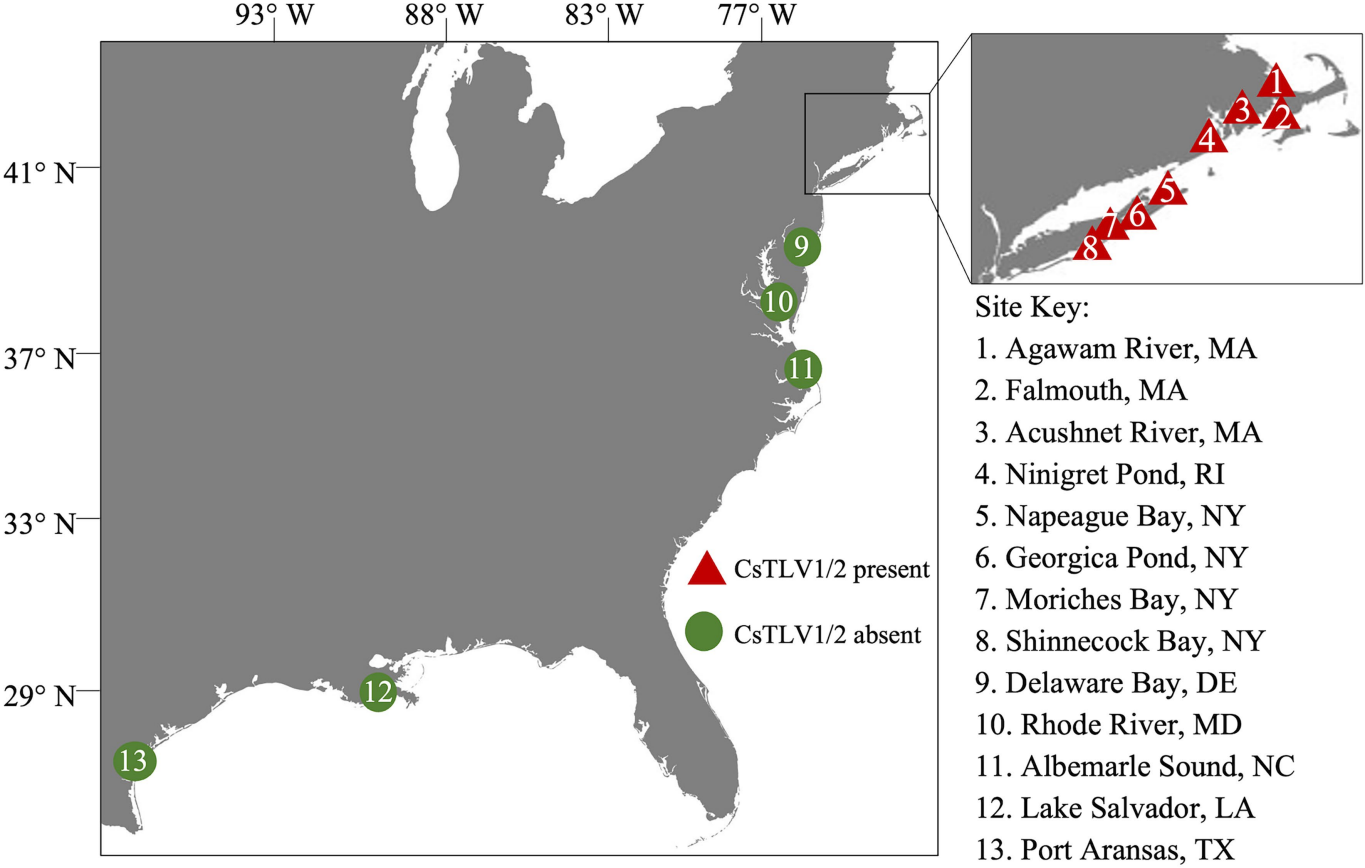
Sampling sites in the northeastern Atlantic coast of the United States. Triangles 1–8 within the inset show sampling sites in MA, RI, and NY. Circles 9–13 indicate sampling sites in DE, MD, NC, LA, and TX. Red color indicates the presence of *Callinectes sapidus* toti-like virus 1 (CsTLV1) and *Callinectes sapidus* toti-like virus 2 (CsTLV2); and green indicates the absence of CsTLV1 and CsTLV2.

Blue crabs were collected from coastal states of the United States, including Massachusetts (MA), Rhode Island (RI), New York (NY), Maryland (MD), Delaware (DE), North Carolina (NC), Texas (TX), and Louisiana (LA) between the years 2009 and 2021 (Figure 1). A portion of crabs collected prior to 2020 were also used in the analysis of CsRV1 prevalence (Zhao et al., 2020). Crab sex and carapace width (CW, measured laterally spine-to-spine), sampling date and locations were recorded during collection. Whole crab or two walking legs removed from each crab were chilled on ice at the time of harvest. For molecular analysis, frozen specimens were then shipped to the Institute of Marine and Environmental Technology (IMET) in Baltimore, MD and stored at −20°C until further analyses. Live crabs from RI and Shinnecock Bay, NY, collected in the year 2021, were shipped chilled to IMET where tissues were collected for virus purification, histology, and electron microscope observations.

### RNA Extraction

Crab dissections were performed with sterile wooden rods and single-use razor blades on a bench cleaned with ELIMINase™. After the external cuticle was cleaned with ELIMINase™, approximately 50 mg of muscle and hypodermis was dissected from a walking leg and homogenized in 1.0 ml of homemade Trizol (Rodriguez-Ezpeleta et al., 2009), with a Savant FastPrep™ FP120 homogenizer (MP Biomedicals, Santa Ana, CA, United States). RNA extraction followed protocols used by Spitznagel et al. (2019). After Trizol-chloroform separation of RNA and precipitation with isopropanol, two 12,000*g* centrifuge washes with 500 μl 75% ethanol were carried out. Resulting RNA pellets were dissolved in 50 μl 1 mM EDTA and stored at −80°C. Process control samples (muscle from frozen smelt) were extracted before and after sets of tested crab samples to monitor for cross contamination between each sample. RNA quality and concentration were determined by NanoDrop™ spectrophotometry (Thermo Scientific, Waltham, MA, United States). The dsRNA content of RNA extractions was revealed by electrophoresis on 1.0% agarose TBE gel and stained with ethidium bromide. The isolated dsRNA was agarose gel purified with the NucleoSpin® Gel and PCR Clean-Up Kit (Takara Bio, San Jose, CA, United States).

### DNA Library Construction and High Throughput Sequencing

Purified dsRNA of a single crab infected with both *Callinectes sapidus* toti-like virus 1 (CsTLV1) and *Callinectes sapidus* toti-like virus 2 (CsTLV2), collected from Agawam River, MA, United States was used for cDNA synthesis with barcoded octamers (5’-GGCGGAGCTCTGCAGATATC-NNNNNNNN-3’) with the M-MLV Reverse Transcriptase 1st-Strand cDNA Synthesis Kit (Biosearch Technologies, Hoddesdon, United Kingdom). The resulting cDNA was amplified by PCR using the barcode primers (5’-GGCGGAGCTCTGCAGATATC-3’). Amplification was achieved through 40 cycles of 95°C for 5 s (denaturation), and 60°C for 30 s (annealing), followed by 72°C for 30 s (elongation). PCR products of 250–500 bp were obtained by agarose gel purification with a NucleoSpin® Gel and PCR Clean-Up Kit (Takara Bio, San Jose, CA, United States). The DNA library was constructed using the NEBNext R Ultra™ DNA Library Prep Kit for Illumina (NEB, Ipswich, MA, United States) following manufacturer’s instructions (NEB, Ipswich, MA, United States). The library was sequenced in a 2 × 250 paired-end configuration on the Illumina MiSeq platform with a MiSeq Reagent kit v3 (Illumina, San Diego, CA, United States).

### Sequence Analyses

Sequencing barcodes were trimmed, and low quality and short reads were removed with CLC Genomics Workbench 9.5.2 (Qiagen, Hilden, Germany). The clean reads were collected and used for *de novo* assembly (Grabherr et al., 2011) with default settings (word-size = 45, Minimum contig length > =500). A preliminary set of contigs coding proteins of at least 150 amino acids were identified with ORF finder in CLC Genomics Workbench. ORFs of *de novo* derived contigs were used to search using the NCBI web server for non-redundant database using the BLASTp program. The conserved domains and motifs in the ORF were searched by NCBI Conserved Domain Database (CDD; http://www.ncbi.nlm.nih.gov/Structure/cdd/wrpsb.cgi). Dotknot (Huang et al., 2005) was used to search the H-type pseudoknots with estimated free energy (EFE). Predicted RNA secondary structures were visualized by Pseudoviewer 2.5 (Byun and Han, 2006).

### Rapid Amplification of cDNA Ends and Sequence Verification

Terminal sequences were determined using a SMARTer® Rapid Amplification of cDNA Ends (RACE) 5′/3′ Kit (Takara Bio, San Jose, CA, United States) with purified dsRNA as the initial template. The 3′ poly(A) tailing of RNA was performed at 37°C for 30 min using *E. coli* Poly(A) Polymerase (NEB, Ipswich, MA, United States). Poly (A)-tailed dsRNA was used for RACE First-strand cDNA synthesis of each terminus using 5′- or 3′- CDS primers as described by the manufacturer (Takara Bio, San Jose, CA, United States). Then, 5′-RACE and 3′-RACE PCR amplification was performed with viral gene specific primers (GSP) and universal primers (UMP), and then a nested PCR was performed with gene specific primers short (GSPS) and universal primer short (UPS; Supplementary Table 1) using Advantage 2 polymerase Mix (Takara Bio, San Jose, CA, United States). The conditions for amplification were 30 cycles at 94°C for 30 s, annealing at 68°C for 30 s, and elongation at 72°C for 2 min, with a final extension at 72°C for 10 min. Amplicons were then purified from the gel using NucleoSpin® Gel and PCR Clean-Up Kit (Takara Bio, San Jose, CA, United States), cloned into pGEM®-T Vector Systems (Promega Corporation, Madison, WI, United States), and sequenced. Sequence verification and filling of gaps between contigs were achieved with Sanger sequencing with primers in Supplementary Table 1. PCR conditions were 30 cycles at 94°C for 30 s, annealing at 56°C for 30 s, and elongation at 72°C for 90 s, with a final extension at 72°C for 10 min.

### Sequence Alignment and Phylogenetic Analyses

Predicted RdRps from all reference sequences, and closest homologues from NCBI were aligned with RdRps of newly identified viruses in this study using MAFFT 7.0 (Katoh and Standley, 2013) with an accurate option (L-INS-i). The alignment was used for constructing the Maximum Likelihood (ML) phylogenetic tree with protein substitution model JTT in CLC Genomics Workbench. RdRp amino acid sequence of *Helminthosporium victoriae* virus 145S (HvV145S; Refseq. YP-052858) was used for the outgroup of the ML tree. Branch support values greater than 0.5 were shown in the tree. The accession numbers of the proteins and the corresponding virus names and acronyms are shown in Supplementary Table 2.

### Reverse Transcription Quantitative PCR Development

To screen CsTLV1 and CsTLV2 infections in *C. sapidus*, a probe-based RT-qPCR assay was developed with primer pairs designed to detect a 193-bp region of CsTLV1 genome and a 183-bp region of CsTLV2 (Table 1) simultaneously. Probes designed for detecting CsTLV1 and CsTLV2 were also shown in Table 1. DsRNA standards were created by *in vitro* RNA synthesis. In brief, PCR products amplified by the primer pairs mentioned above were purified and cloned into pGEM®-T Vector Systems (Promega Corporation, Madison, WI, United States). Plasmids containing the targeted region were used as templates to synthesize each strand of the viral RNA standards by T7 or Sp6 RNA polymerase, respectively (Sigma-Aldrich, St. Louis, MO, United States). Viral RNAs were then quantified and annealed into dsRNA on ice, and serially diluted in 25 ng per μl yeast tRNA carrier. Standard curves were generated by RT-qPCR amplifications of a 10-fold dilution series of synthesized dsRNA containing 10–10e6 genome copies per μl. The qPCR cycling contained qScript® Virus 1-Step ToughMix® (Quantabio, Beverly, MA, United States) in 10 μl reactions with 0.25 μM each primer and 0.25 μM each probe for both genomes. To anneal PCR primers to dsRNA, primers and extracted RNA were combined, heated to 95°C for 5 min, and then cooled to 4°C prior to being added to the reverse transcriptase and Taq polymerase reaction mixture. Reverse transcription and amplification conditions were 50°C for 10 min (reverse transcription) followed by 1 min at 95°C (reverse transcriptase inactivation and template denaturation). Amplification was achieved through 40 cycles of 95°C for 10 s, and 61°C for 30 s. Gene target copies were then calculated as copies per mg of crab muscle, and samples with greater than 100 copies per mg were recorded as CsTLV1 and CsTLV2 positive, which was based on empirical observations of cross contamination in process control RNA extractions.

**Table 1:** Primers and probes used in reverse transcription quantitative PCR (RT-qPCR).

Name		Sequences	Size (bp)
CsTLV1	forward	5’-GCAAAGGAGTGAAGGAGTGG-3’	193
	reverse	5’-GCAAGACGCATAGCACGATA-3’	
	Probe	5’6-FAM/TGCTTGCGG/ZEN/AGAAACTGAACGAGA/3’IABkFQ	
CsTLV2	forward	5’-ACGGCGACTTTGTTGAGT TT-3’	183
	reverse	5’-ACGGTAACCCAGACCATTGA-3’	
	probe	5’Cy5/AGTTGGGAG/TAO/GCAGAGATGTGTGTT/3’IAbRQSp	

### Statistical Analyses

All statistical tests were conducted using RStudio 1.1.456 (R Core Team, 2019). Significant correlations were defined as those where *p* ≤ 0.05. To determine whether CsTLV1 and CsTLV2 infections were correlated with sex, crab size or latitude, binomial (infected vs. non-infected) generalized logistic regression models (GLM) were conducted (alpha = 0.05). Akaike’s information criterion (AIC) was used to choose the best GLM to determine, which factors best correlate with CsTLV1 and CsTLV2 infection (Aho et al., 2014). The Pearson correlation was used to test the correlation between the variables (Kirch, 2008).

### Histology and Electron Microscopy

*Callinectes sapidus* collected from RI and Long Island (NY) with CsTLV1 and CsTLV2 confirmed and quantified by RT-qPCR were dissected with muscle, gill and hepatopancreas tissues removed for further examination for virus presence. These crabs were also tested by RT-qPCR (methods in Zhao et al., 2020) to confirm they were not infected by CsRV1. For histological analyses, the tissues were fixed in Bouin’s solution at 4°C overnight, and then placed into 75% ethanol for long-term storage. Preserved tissues were processed according to the standard operating procedures for embedding, sectioning and Hematoxylin and Eosin (H&E) staining (Luna, 1968). Slides were then observed with an Echo Revolve Microscope (San Diego, CA, United States).

For electron microscopy examination, crab tissues were immersion fixed in fixative buffer (2% paraformaldehyde, 2.5% glutaraldehyde, 2 mM CaCl2 in 0.1 M PIPES Buffer, and pH 7.35) at 4°C overnight. Tissue fragments were then trimmed into ~1 mm^3^ cubes, post-fixed with 1% osmium tetroxide, washed in water and stained *en bloc* with 1% (w/v) uranyl acetate for 1 h. Specimens were then washed and dehydrated using 30, 50, 70, 90, and 100% ethanol in series. After dehydration, specimens were embedded in Araldite-Epoxy resin (Araldite, Embed 812, Electron Microscopy Sciences, Hatfield, PA, United States; Vorimore et al., 2013). Ultrathin sections (~ 70 nm) were cut and examined in a Tecnai T12 TEM (FEI, Hillsboro, OR, United States) operated at 80 KV. Digital images were acquired using an AMT bottom mount CCD camera and AMT600 software (Advanced Microscopy Techniques, Woburn, MA, United States). Crab samples infected with only CsTLV2 were not preserved well enough to be included in TEM and histology examinations.

## Results

### Detection of Putative Viral dsRNA

**Figure 2:**
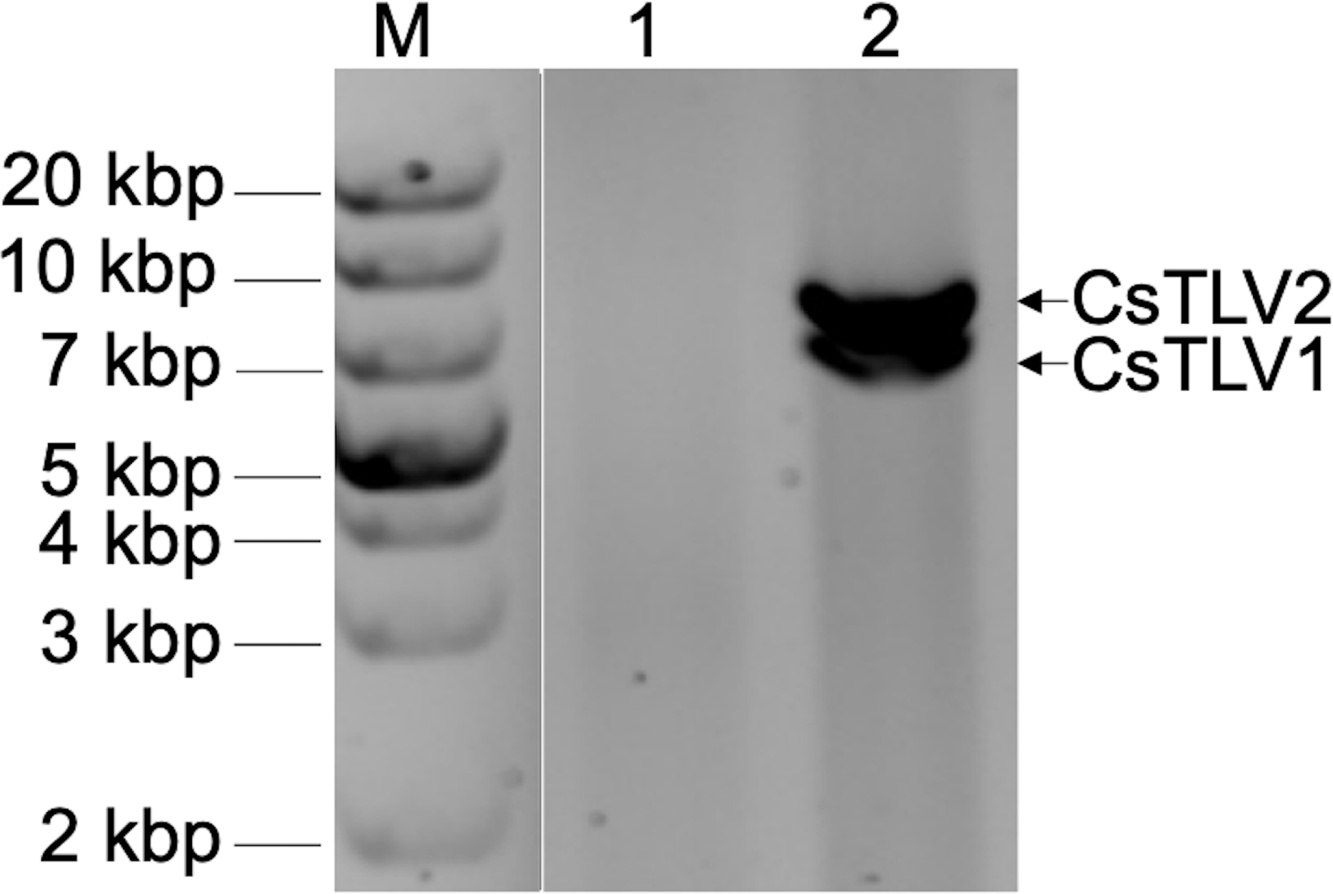
Agarose gel electrophoresis of apparent viral double-stranded RNA (dsRNA) bands of CsTLV1 and CsTLV2. M, marker; Lane 1, control RNA of uninfected blue crab leg muscle; and Lane 2, RNA extracted from infected blue crab leg muscle showing two dsRNA bands of dsRNA-S and dsRNA-L, respectively.

In a search for viral dsRNA in blue crabs, RNA extracted from leg muscle analyzed on agarose gels. The RNA of 31% of sampled of crabs (9 of 29) harvested from the Agawam River, MA in the summer of 2008, showed two prominent dsRNA bands, termed dsRNA-S and dsRNA-L. The apparent molecular weights of the two dsRNA segments were ∼6.5 and ∼7.5 kbp, respectively (Figure 2).

### Sequence Analyses of dsRNA-S and dsRNA-L

**Figure 3:**
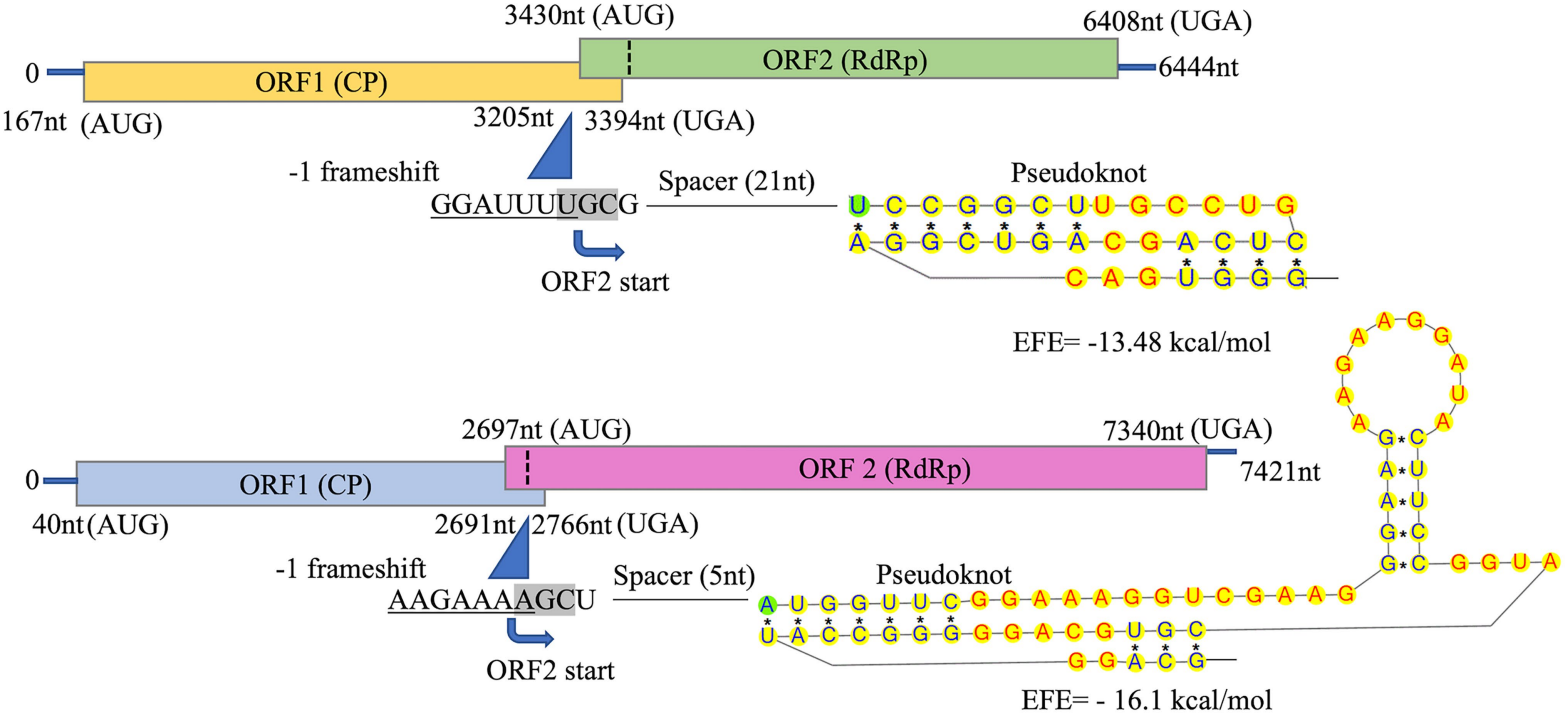
Schematic representation of CsTLV1 and CsTLV2 genome. The two overlapping open reading frames (ORFs) and the untranslated regions (UTRs) are shown by boxes and a single line, respectively. Nucleotide positions of ORFs and the putative slippery site for −1 frameshifting, spacer and pseudoknot are indicated too. EFE (kcal/mol) indicates the minimal free energy.

Total dsRNA, containing both dsRNA-S and dsRNA-L was sequenced with NGS. Assembly of trimmed and quality filtered reads (16,650 reads: 42.4% of the total reads) resulted in two contigs for each CsTLV1 and CsTLV2, at 383- and 120-fold average coverage, respectively. The longest contig was 4,016 nucleotides (nt) in length. The 5′ and 3′ untranslated regions (UTRs) of both genomes were obtained by RACE PCR and Sanger sequencing, to reveal two toti-like genome sequences of 6,444 and 7,421 nt, and designated as CsTLV1 and CsTLV2, respectively. Each genome contained two ORFs (ORF1 and ORF2) encoding Cp and RdRp proteins, respectively (Figure 3). Genomic sequences were not detected for any other virus in the NGS library.

**Figure 4:**
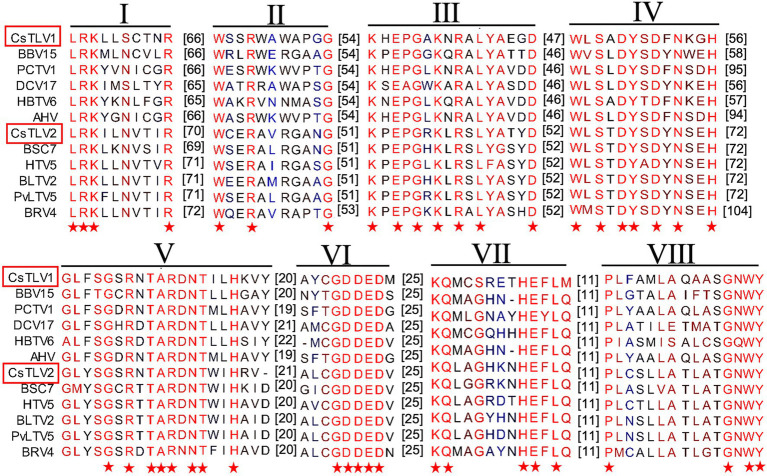
Conserved motifs in RNA dependent RNA polymerase protein (RdRp) of CsTLV1 and CsTLV2. Amino acid sequences alignment of CsTLV1 and CsTLV2 with closely related toti-like viruses from NCBI database. Horizontal lines above the alignment indicate the eight motifs, numbers in brackets indicate the amino acid sequence lengths between the motifs, asterisks indicate identical amino acid residues, and color gradients indicate the similarity level of amino acid residues. Virus notations are as in Supplementary Table 2.

The predicted Cp and RdRp proteins of CsTLV1 and CsTLV2 showed limited amino acid sequence identity with each other (21% for Cp and 27% for RdRp, respectively). CsTLV1 RdRp amino acid sequences showed >33% identity with the corresponding predicted RdRp of Beihai barnacle virus 15, Ahus virus, Parry’s Creek toti-like virus 1, and Diatom colony associated dsRNA virus 17 genome type A (Shi et al., 2016; Urayama et al., 2016; Pettersson et al., 2019; Williams et al., 2020). CsTLV2 RdRp amino acid sequence showed >40% identity with the RdRp encoded in *Plasmopara viticola* lesion associated totivirus-like 5, Hubei toti-like virus 5, and Beihai sesarmid crab virus 7 (Shi et al., 2016; Chiapello et al., 2020; Supplementary Table 3). A search of the CDD and multiple protein alignment confirmed that the predicted RdRp domains of CsTLV1 and CsTLV2 contain eight conserved motifs (I–VIII), including the GDD motif, which are the typical characteristics of virus RdRps (Figure 4). Sequence analyses of CsLTV1 and CsTLV2 indicated that there is an overlap region between ORFs 1 and 2 (Figure 3), that allows ORF 2 to be translated as a fusion protein with ORF 1 through a-1 ribosomal frameshift motif “GGAUUUU” at 3,199–3,205 nt positions in CsTLV1, and “AAGAAAA” at positions of 2,685–2,691 nt in CsTLV2. An H-type pseudoknot structure was predicted in the downstream of each putative slippery site at positions 3,227–3,259 nt of CsTLV1 genome and 2,697–2,760 nt of CsTLV2 (Figure 3).

### Phylogeny of CsTLV1 and CsTLV2

**Figure 5:**
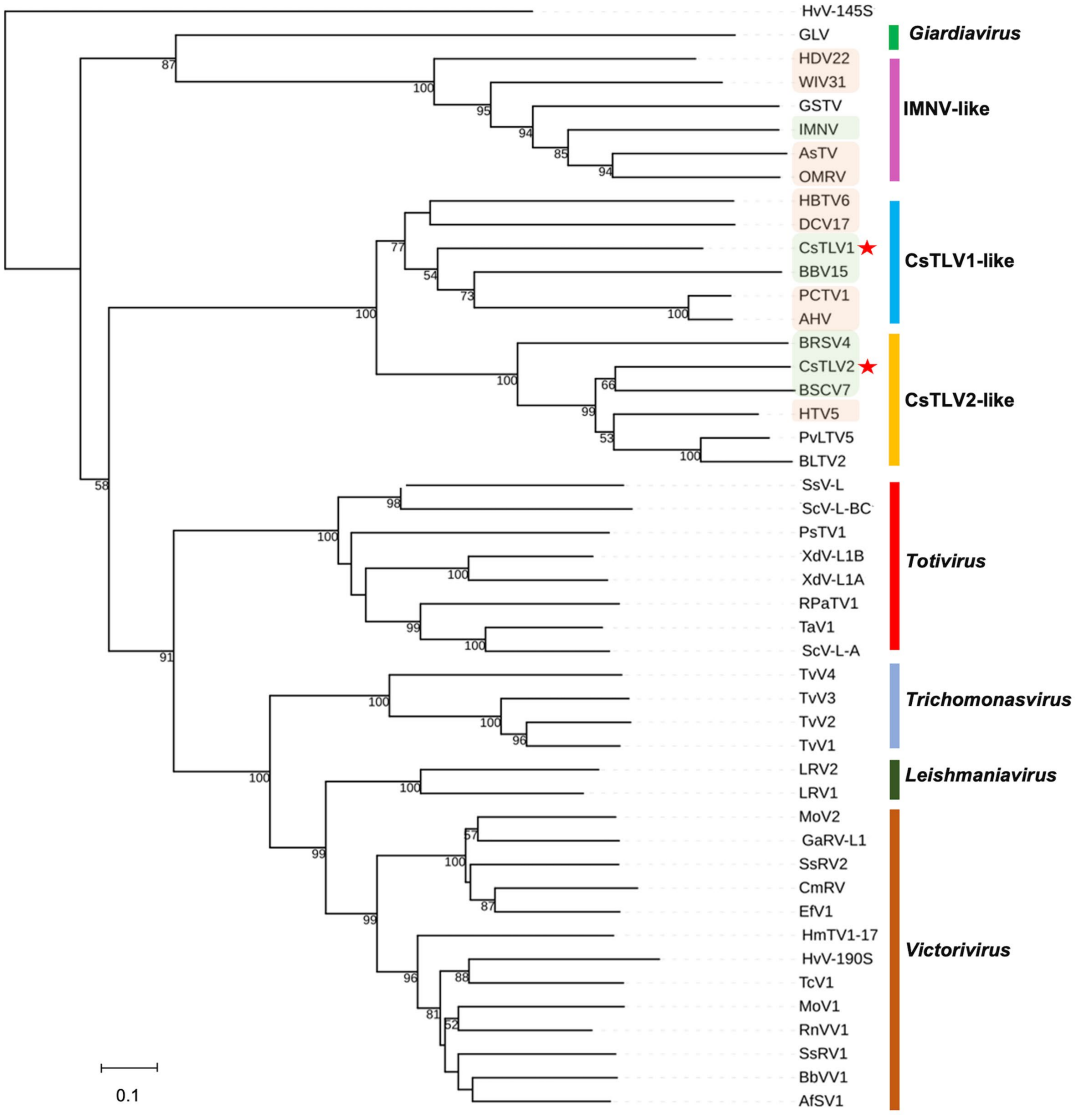
Phylogenetic relationships between putative RdRp amino acids of CsTLV1 and CsTLV2 with other selected *Totiviridae* members. A phylogenetic tree was generated using the maximum likelihood (ML) method with 1,000 bootstrap replicates. CsTLV1 and CsTLV2 are highlighted with asterisks. Green shade indicates totiviruses identified in crustacean hosts and orange shade indicates totiviruses in other arthropod hosts. Virus notations are as in Supplementary Table 2.

A maximum likelihood phylogenetic tree was used to show the relationships between CsTLV1 and CsTLV2 and other selected totivirids. As shown in Figure 5, RdRp amino acid sequence multiple alignments of CsTLV1 and CsTLV2 and the corresponding toti and toti-like viral sequences revealed that CsTLVs is most closely related to, but distinct from, *Totivirus*, and Artivirus which is a proposed genus that includes IMNV and IMNV-like viruses (Zhai et al., 2010). CsTLV1 and the five toti-like viruses with the highest identity from GenBank formed a cluster in the tree (bootstrap value = 77%), adjacent to but different from the cluster of CsTLV2 and its close toti-like virus species (bootstrap value = 100%).

### RT-qPCR Assay Performance

The probe-based RT-qPCR assay consistently detected as few as 10 copies of the target when tested on a dilution series of synthesized dsRNA standards (Table 2). Efficiency and sensitivity of the assay were evaluated by running10 RT-qPCR standard curves. The mean slope was 3.29 with a SD of 0.06 for CsTLV1, and the mean slope was 3.25 with a SD of 0.07 for CsTLV2. There is an average efficiency of 100.1 and 100.3% for CsTLV1 and CsTLV2 respectively, under typical use with the synthesized dsRNA standard.

**Table 2:** RT-qPCR efficiency with dsRNA standards.

Genome	Slope	*Y*-intercept	*R* ^2^	Efficiency
CsTLV1	3.29	38.730	0.995	100.1
CsTLV2	3.25	37.624	0.995	100.3

*The threshold cycles for a log10 dilution series are used to assess efficiency relative to 100% theoretical efficiency for a slope of 3.32 based on 10 replicates*.

### Prevalence of CsTLV1 and CsTLV2 Infections and Co-infections

The prevalence of CsTLV1 and CsTLV2 was investigated by RT-qPCR in 875 crabs from the US Atlantic and Gulf of Mexico coasts. CsTLV1 and two infections were detected in the northern states (MA, RI, and NY, *n* = 496) but not the lower latitudinal Atlantic states (DE, MD, and NC, *n* = 299) and Gulf states (LA and TX, *n* = 80). CsTLV1 and CsTLV2 infections in *C. sapidus* were detected in all three estuaries sampled in MA: Agawam River, Acushnet River, and Falmouth (Figure 1; Table 3). CsTLV1 RNA prevalence in *C. sapidus* sampled from MA (*n* = 198) varied from 11.8% (Agawam River; *n* = 127) to 30.6% (Acushnet River; *n* = 49), and CsTLV2 RNA prevalence ranged from 12.6 to 36.7%. In crabs from RI, CsTLV1 prevalence was 27.6% and CsTLV2 prevalence was 32.8%. Viral RNA was detected in crab specimens collected from Moriches Bay, Shinnecock Bay, and Napeague Bay in Long Island, NY (*n* = 133) with an average prevalence of 42.1 and 43.6% for CsTLV1 and CsTLV2, respectively. CsTLV1 RNA was detected in Georgica Pond, NY, with low prevalence in 2012 (5.5%; *n* = 18), but not in 2013, 2020, or 2021, and CsTLV2 RNA was detected in 2012 (5.5%; *n* = 18) and 2013 (5.2%; *n* = 19), but not in 2020 (*n* = 33) or 2021 (*n* = 37).

**Table 3:** CsTLV1 and CsTLV2 prevalence in *C. sapidus*. Specimens were collected from locations along the US Atlantic coasts and Gulf coasts of the United States.

Location	Collection date (Month-Year)	Latitude	Longitude	Total *N*	CsTLV1		CsTLV2	
					Infected	Pre	Infected	Pre
					(*N*)	(%)	(*N*)	(%)
Agawam River, MA	Aug-2009	41.7619°N	71.6773°W	29	11	37.9	11	37.9
	Aug-2012			47	4	8.5	4	8.5
Falmouth, MA	Sep-2018	41.5388°N	70.6266°W	51	0	0	1	2
	Sep-2018			22	4	18	4	18
Acushnet River, MA	Aug-2012	41.6617°N	70.9182°W	49	15	30.6	18	36.7
Rhode Island, RI	Aug-2021	41.3697°N	71.6426°W	58	16	27.6	19	32.8
Napeague Bay, NY	Jul-2021	40.9987°N	72.0972°W	10	6	60	6	60
Georgica Pond, NY	Aug-2012	40.9361°N	72.2138°W	18	1	5.5	1	5.5
	Jul-2013			19	0	0	1	5.2
	Jul-2020			33	0	0	0	0
	Jul-2021			37	0	0	0	0
Moriches Bay, NY	Jul-2018	40.7738°N	72.8052°W	32	7	21.8	8	36.7
	Jul-2021			25	4	16	5	20
Shinnecock Bay, NY	Jul-2021	40.8426°N	72.4762°W	28	18	64.3	18	64.3
	Sep-2021			21	10	47.6	10	47.6
				17	11	64.7	11	64.7
Delaware Bay, DE	Apr-2019	38.9108°N	75.5277°W	51	0	0	0	0
	Aug-2021			38	0	0	0	0
Rhode River, MD	Mar-2015	38.8795°N	76.5216°W	33	0	0	0	0
	Jul-2018			52	0	0	0	0
	Aug-2020			30	0	0	0	0
Albemarle Sound, NC	Oct-2019	33.8772° N	76.1248° W	95	0	0	0	0
Port Aransas, TX	Jan-2021	27.8339° N	97.0611° W	40	0	0	0	0
Lake Salvador, LA	Jan-2021	29.7192° N	90.2432° W	40	0	0	0	0

*Pre: Prevalence*.

Overall, CsTLV1 was never observed in the absence of CsTLV2, and co-infections of CsTLV1 were detected in 91.5% (107/117) of CsTLV2 positive specimens (Table 3). The dsRNA copy number per mg muscle ranged from 6.5 × 10e2 to 1.2 × 10e8 for CsTLV1, and 1.3 × 10e2 to 6.3 × 10e8 for CsTLV2.

### Correlation of CsTLV1 and CsTLV2 Infection With Latitude and Crab Size

A binomial (infected vs. non-infected) generalized linear model (GLM) was used to test whether latitude, crab size, or sex could predict CsTLV1 and CsTLV2 infection status (Table 4). The 415 male and 140 female specimens, from 20 to 196 mm in carapace width (CW), were used for GLM analysis. Specimens that were PCR-positive for CsTLV1 and CsTLV2 ranged from 29 to 150 mm in CW. Prevalence for male and female crabs was 12.0 and 10.7%, respectively. Pearson correlation tests showed no significant correlation between latitude and crab size or sex. The full model analyzing the association between CsTLV1 and CsTLV2 infections and latitude, crab sex, and carapace width (CW), differed significantly from null models (*p* < 0.01), in which latitude and CW were significant fixed effects (*p* < 0.01). The reduced model, including only the association between CsTLV1 and CsTLV2 infection and latitude, sex, or CW, reinforced that latitude and CW were the significant factors correlated with CsTLV1 and CsTLV2 prevalence (*p* < 0.01; Table 4). CsTLV1 and CsTLV2 prevalence was positively related to latitude in the reduced model (slope is 2.33 for CsTLV1 and 2.38 for CsTLV2; *p* < 0.01), and CW showed a negative association with CsTLV1 and CsTLV2 prevalence (slope = −1.00 for CsTLV1 and slope = −1.13 for CsTLV2; *p* < 0.01). In both full and reduced models, the association between CsTLV1/CsTLV2 prevalence and crab sex was not significant (*p* > 0.1).

**Table 4:** Generalized linear model (GLM) with potential effects on CsTLV1 and CsTLV2 infection.

Model	Predictor variable	Estimate (slope)	Standard Error	*p*-value
A. Full model:				
CsTLV1 Infection~ Latitude + Size + Sex (AIC = 339.98; df = 551)	Latitude	2.54	0.64	6.64e−05^***^
	Size	−1.00	0.25	9.47e−05^***^
	Sex	−0.31	0.35	0.37
CsTLV2 Infection1 ~ Latitude + Size + Sex (AIC = 340.89; df = 551)	Latitude	2.68	0.66	4.77e−05^***^
	Size	−1.12	0.26	1.12e−05^***^
	Sex	−0.30	0.35	0.39
B. Reduced model:				
CsTLV1 Infection ~ Latitude (AIC = 352.24; df = 554)	Latitude	2.33	0.54	1.43e−05^***^
CsTLV1 Infection ~Size (AIC = 382.85; df = 553)	Size	−1.13	0.24	2.24e−06^***^
CsTLV1 Infection ~Sex (AIC = 404.68; df = 553)	Sex	−0.13	0.31	0.67
CsTLV2 Infection ~ Latitude (AIC = 357.72; df = 554)	Latitude	2.38	0.54	9.78e−06^***^
CsTLV2 Infection ~Size (AIC = 385.34; df = 553)	Size	−1.25	0.24	1.42e−07^***^
CsTLV2 Infection ~Sex (AIC = 412.8; df = 553)	Sex	−0.08	0.30	0.78

*The model has the lowest Akaike’s information criterion (AIC) of all combinations of predictor variables (see as full and reduced interactions)*.

*** *denotes significance (*p* < 0.001)*.

### Electron Microscopy: Observation of Viral Particles

**Figure 6:**
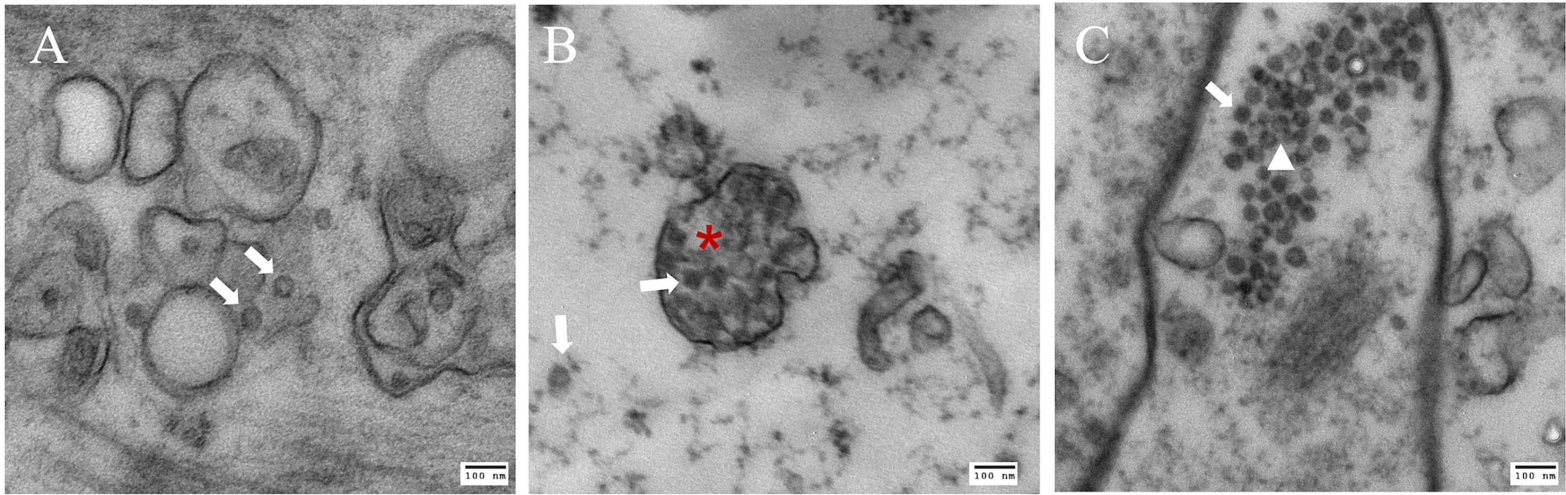
Electron microscopy images of putative CsTLV1 and CsTLV2 viral particles in muscle, gill, and hepatopancreas tissues of *Callinectes sapidus*. **(A)** muscle; **(B)** gills; and **(C)** hepatopancreas. White arrow: virions; Red star: putative “viroplasm” in gill. White triangle: dense arrangement of virions in hepatopancreas. Scar bar, 100 nm.

Crabs assessed to be infected with CsTLV1 and CsTLV2 by RT-qPCR were selected for TEMs observation (Supplementary Figure 1). TEM revealed the presence of isometric virus particles, with a diameter of ~40 nm in *C. sapidus* muscle, gill, and hepatopancreas tissues (Figure 6). Completed virions were present in the connective tissue and hemocytes of these tissues. We observed putative viroplasm in the gill of CsTLV1 and CsTLV2 infected crab and packed arrays of mature virions in the hepatopancreas of infected crab.

### Histopathology of CsTLV1 and CsTLV2 Co-infected Blue Crab Tissues

**Figure 7:**
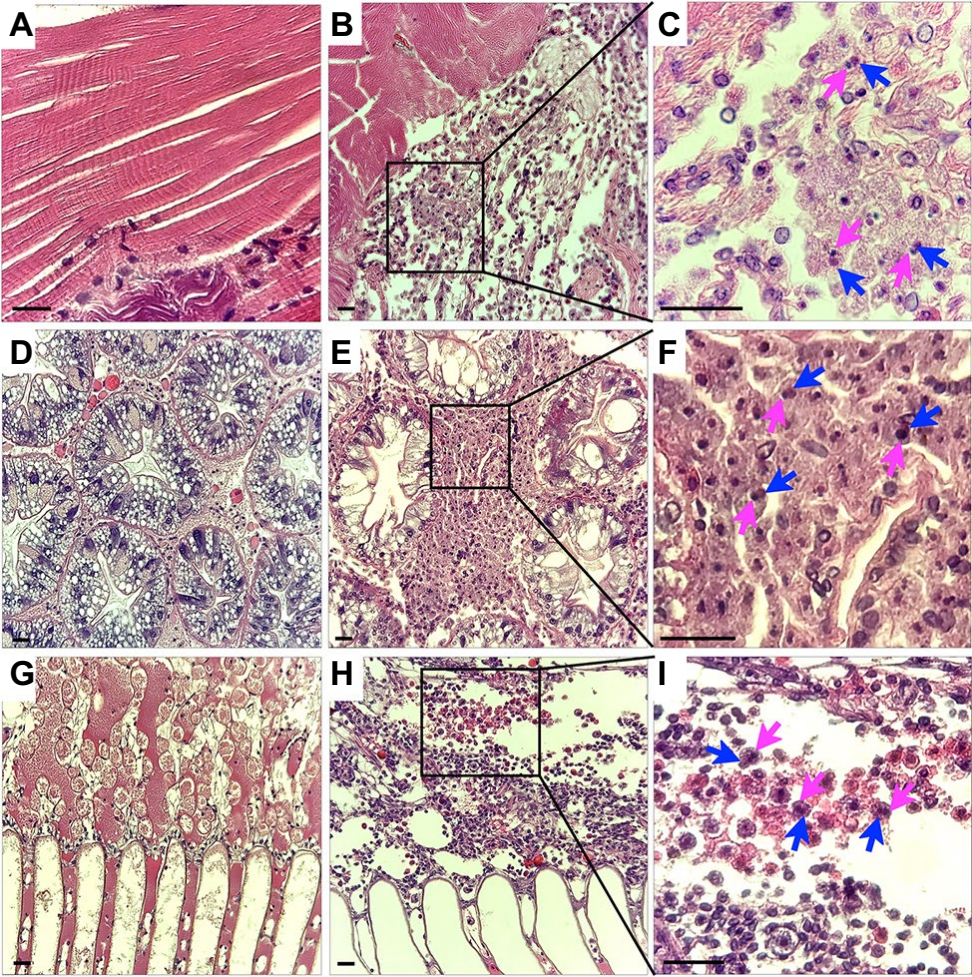
Histology of CsTLV1 and CsTLV2 infections in muscle, gill, and hepatopancreas of *Callinectes sapidus*. **(A,D,G)** Muscle, hepatopancreas, and gill of uninfected crabs; **(B,E,H)** Muscle, hepatopancreas, and gill of CsTLV1 and CsTLV2 infected blue crabs; **(C,F,I)** Magnified view of boxed area in **(B,E,H)**, respectively. Infected hemocytes in muscle, hepatopancreases, and gill often had pyknotic or karyopyknotic nuclei (magenta arrows) as well as opaque, slightly eosinophilic intracytoplasmic inclusion bodies (blue arrows). Scale bar, 25 μm.

Histological analysis of muscle, hepatopancreas and gills tissues of crabs naturally infected with CsTLV1 and CsTLV2 showed necrosis and hemocyte infiltration. Skeletal muscle in normal uninfected crabs is generally smooth, striated and with few circulating hemocytes (Figure 7A). Infected skeletal muscle had general necrosis and showed vacuolated areas with increased numbers of circulating hemocytes (Figure 7B). Hepatopancreas tubules in normal uninfected crabs have defined outer membranes and moderate numbers of circulating hemocytes circulating within hemal spaces between tubules (Figure 7D). Infected hepatopancreas often showed massive hemocytic infiltration (Figure 7E). Gills of normal uninfected crabs have moderate numbers of circulating hemocytes in hemal spaces (Figure 7G). Infected gills had considerably increased numbers of circulating hemocytes within necrotic areas (Figure 7H). At higher magnification, infected hemocytes in muscle, hepatopancreas, and gills often had pyknotic or karyorrhectic nuclei (magenta arrows) as well as opaque, slightly eosinophilic intracytoplasmic inclusion bodies (blue arrows; Figures 7C,F,I).

## Discussion

Molecular approaches for discovery of virus-like genomes have verified that viruses are an important and universal feature of the life history of marine organisms (Munn, 2006; Suttle, 2007). Beyond the discovery of new viruses, the characterization of these newly discovered viruses contributes to better understanding of their diversity, evolution, and ecology in marine environments. Partial toti-like virus sequences, reported in some crabs by metagenomics (e.g., in Shi et al., 2016), have only documented virus-like genome elements, but not the presence of virus particles. In this study, we sequenced and characterized the genomes of two new putative *C. sapidus* totiviruses—CsTLV1 and CsTLV2, and showed that viral particles are present in tissues of CsTLV1 and CsTLV2 co-infected crabs and are associated with pathology. This study is the first description of an endemic infection of totivirus in *C. sapidus*.

Both CsTLV1 and CsTLV2 genomes contained two ORFs encoding the conserved domains of Cp and RdRp, respectively. Moreover, the two viruses contain a-1 ribosomal frameshifting in their genomes (Figure 3), which could facilitate the translation of ORF1 and ORF2 as a fusion polyprotein (Dinman et al., 1991). The predicted ORF2 coding strategy of CsTLV1 and CsTLV2 was consistent with other viruses in the family *Totiviridae*, such as *Saccharomyces cerevisiae* virus L-A (ScVL-A; Dinman et al., 1991) and IMN virus (IMNV; Nibert, 2007). CsTLV1 and CsTLV2 have all three elements that are required to accomplish −1 ribosomal frameshifting in RNA viruses: a slippery heptamer motif, an RNA pseudoknot shortly downstream of the site and a short spacer region between the slippery site and the pseudoknot (Rice et al., 1985; Dinman et al., 1991; Khalifa and MacDiarmid, 2019). The classical slippery site sequence is “XXXYYYZ” (where X is A/C/G/U, Y is A/U, and Z is A/C/U) within the overlapping region (Bekaert and Rousset, 2005). The slippery site of CsTLV1 (GGAUUUU) is the same to the slippery heptamer nucleotides found in other totiviruses, such as *Xanthophyllomyces dendrorhous* viruses (GGAUUUU; Baeza et al., 2012), *Puccinia striiformis* totiviruses (PsTVs; GGG/AUUUU; Zheng et al., 2017) and red clover powdery mildew-associated totiviruses (RPaTVs; GGG/AUUUU; Kondo et al., 2016). Meanwhile, the slippery site is “AAGAAAA” in CsTLV2, which is the same as that used by plant associated astro-like virus (Lauber et al., 2019).

In the current ICTV scheme of totivirus taxonomy, 50% sequence identity of Cp/RdRp proteins is generally considered a threshold to define different species (Wickner et al., 2011). CsTLV1 and CsTLV2 share only 21% identity for Cp and 27% for RdRp, indicating they are distinct species in the family *Totiviridae*. Phylogenetic analyses of RdRp amino acid sequences showed that CsTLV1 and CsTLV2 formed a distinct branch from other genera in the family *Totiviridae* but clustered into two subgroups (Figure 5). CsTLV1, together with toti-like viruses identified from arthropod and crustacean hosts were classified into one group (Shi et al., 2016), and CsTLV2 formed another group with totiviruses sequenced from spirurian nematodes, sesarmid crab, and razor shell clam *Ensis magnus* (Shi et al., 2016). Compared to other genera of the family *Totiviridae*, members of CsTLV1-like and CsTLV2-like groups have the highest similarity between each other. Taken together with their genome structure and phylogenetic position, CsTLV1 and CsTLV2 may represent two new viral species within two novel genera of the family *Totiviridae*.

Co-infection by two distinct viruses has been reported in *C. sapidus* such as reovirus and RhVA (Johnson, 1978, 1983), and bunya-like virus (Zhang et al., 2004). Co-infection of distinct totiviruses has also been commonly reported, such as in *Sphaeropsis sapinea* and *Chalara elegans* (Preisig et al., 1998; Park et al., 2005). Recently, co-infection of three dsRNA viruses *Trichomonas vaginalis* virus (TVV1, TVV2, and TVV3) were revealed (Bokharaei-Salim et al., 2020). Co-infection of CsTLV1 was detected in more than 90% CsTLV2-positive specimens (Table 3), suggesting that there is a significant relationship between these two totiviruses in *C. sapidus*. Interestingly, although independent infection of CsTLV2 was identified, no crab was ever found that contained the CsTLV1 genome alone. One possible explanation for this observation may be that the CsTLV1 genome or virus cannot replicate or be encapsulated in the absence of CsTLV2. The relationship between CsTLV1 and 2 does not have the characteristics of defective virus genomes (Vignuzzi and López, 2019); the CsTLV1 genome does not have obvious deletions or frame shifts, although the CsTLV2 genome is over 1,000 nt longer than the CsTLV1 genome. A similar phenomenon has been revealed that *Helminthosporium victoriae* virus 190S (HvV190S; *Totiviridae*) and *Helminthosporium victoriae* virus 145S (HvV145S; *Chrysoviridae*) co-infect the pathogenic fungus *Helminthosporium victoriae*. HvV145S has never been found alone but is always associated with HvV190S virus. HvV145S was originally thought to be the cause of the diseases, however, a recent study suggested that HvV190S alone is the cause of diseases, and the co-infection is not required (Xie et al., 2016). In our study, TEM of co-infected blue crabs revealed all virions had a diameter of ~40 nm, suggesting that either CsTLV1 is indistinguishable in size or appearance from CsTLV2, or that only one of the viruses produces virions.

Most members of *Totiviridae* infecting fungi and protozoans lack extracellular transmission; instead, they are transmitted vertically during cell division, sporogenesis, and cell fusion (Ghabrial and Suzuki, 2009). However, some totiviruses with fiber-like protrusions on their surface, such as IMNV and Omono River virus (OmRV), are capable of extracellular transmission in their metazoan hosts (Poulos et al., 2006; Tang et al., 2008; Dantas et al., 2015; Shao et al., 2021). The transmission mechanism for CsTLV1 and CsTLV2 in the blue crab is yet unknown. Attempts to transmit the viruses by injection of previously frozen material (CsTLV1 and CsTLV2) into naïve crabs have been so far unsuccessful (Zhao and Schott, unpublished data). Necrosis and massive hemocyte infiltration in CsTLV1 and CsTLV2 infected muscle, gill, and hepatopancreas suggested that the viruses are detrimental to the health of blue crabs. CsTLV1 and CsTV2 infections were negatively correlated with crab size in GLM analyses, which suggested that juveniles may be more susceptible to infection, or that older animals infected with CsTLV1 and CsTLV2 either die or clear the virus as they mature or age. All these results provide the fundamental knowledge for future studies to investigate how these viruses are transmitted and how they affect the ecology of blue crabs.

The significant correlation between CsTLV1 and CsTLV2 infections and latitude has also been identified in another blue crab dsRNA virus-CsRV1, which also showed significantly higher prevalence at higher latitudinal locations compared to lower latitudes (Flowers et al., 2016, 2018; Zhao et al., 2020). However, compared to the wide geographic range of CsRV1 infections in blue crabs, infections of CsTLV1 and CsTLV2 were restricted to the most northeastern estuaries we sampled in MA, RI, and NY, but absent from the lower latitudinal estuaries of DE, MD, NC, LA, and TX. Although factors driving the emergence of viruses and the gradient of virus prevalence at different geographic locations could be complex, two likely covariates in our study are water temperature and length of the active period for blue crabs, which have strong correlations to latitudes (Zhao et al., 2020). It is notable that the virus is present in crabs at the northern edge of their geographic range. Microbiome community changes and emergence of novel pathogens have been widely reported during the dispersal of host invasion and extension range (Engering et al., 2013; Dragičević et al., 2021). The extensive poleward expansion of *C. sapidus* in its native range along the western Atlantic and its successful invasion to European waters (Johnson, 2015; Mancinelli et al., 2021), made *C. sapidus* a well-suited model to study virus evolution, diversity, and viral ecology of marine animals during host habitat expansion and invasion. In Rhode Island, state managers are beginning to survey blue crab abundance in anticipation of a growing commercial and recreational fishery (K. Rodigue, personal communication). Therefore, further systematic and comprehensive studies on the virome of *C. sapidus*, including CsTLV1 and CsTLV2, at different geographical locations are urgently needed for a better understanding of the virus ecology and epidemiology with the host habitat expansion.

In conclusion, two putative viral dsRNA sequences in *C. sapidus* were characterized with NGS, and shown to be associated with virus particles and histopathology. Based on their genomic organizations, phylogenetic relationships, and conserved motifs, the viruses are tentatively named CsTLV1 and CsTLV2, and proposed to be members of two new genera in the family *Totiviridae*.

## Data Availability Statement

The datasets presented in this study can be found in online repositories. The names of the repository/repositories and accession number(s) can be found at: https://www.ncbi.nlm.nih.gov/genbank/, OL456199, OL456200.

## Author Contributions

ES and MZ designed the experiments and analyzed the results and drafted the manuscript. MZ, LX, and HB performed the experiments. ES, MZ, LX, and HB revised the paper. All authors contributed to the article and approved the submitted version.

## Funding

This work was supported by NSF Division of Ocean Sciences—Biological Oceanography awards 1658466 (ES), Maryland Sea Grant—Graduate student research support grant (ES, MZ). MZ and LX were supported by awards from the China Scholarship Council.

## Conflict of Interest

The authors declare that the research was conducted in the absence of any commercial or financial relationships that could be construed as a potential conflict of interest.

## Publisher’s Note

All claims expressed in this article are solely those of the authors and do not necessarily represent those of their affiliated organizations, or those of the publisher, the editors and the reviewers. Any product that may be evaluated in this article, or claim that may be made by its manufacturer, is not guaranteed or endorsed by the publisher.
