# Supplementary material for: Characterization of Two Novel Toti-Like Viruses Co-infecting the Atlantic Blue Crab, Callinectes sapidus, in Its Northern Range of the United States
Source: Front Microbiol. 2022 Mar 3;13:855750. doi: 10.3389/fmicb.2022.855750 (PMC8973213; doi:10.3389/fmicb.2022.855750)
Supplement: Supplementary file 1 [file Table_1.DOCX]

[**Supplementary Table S1**](https://www.frontiersin.org/articles/10.3389/fmicb.2019.00626/full#SM1)**:** Primers for RACE and Sanger sequencing:

| **Name** | **Sequence** |
| --- | --- |
| UMP | 5’-CTAATACGACTCACTATAGGGCAAGCAGTGGTATCAACGCAGAGT-3' |
| UPS | 5’-CTAATACGACTCACTATAGGGC-3’ |
| SMART II™ A Oligonucleotide | 5’-AGCAGTGGTATCAACGCAGAGTACATGGG-3’ |
| CsTLV1 RACE |  |
| 3’-GSP | 5’-CGGCGTGCTGAAGCTAATCGTGTCT-3’ |
| 3’-GSPS | 5’-GGAAGGGTCATGGAGATGGTGGACAGA-3’ |
| 5’-GSP | 5’-GGCACCACATCACGACCACGACAA-3’ |
| 5’-GSPS | 5’-GGCTGCCAGACGCCACTCAAATCTAC-3’ |
| CsTLV2 RACE |  |
| 3’-GSP | 5’-GGA ACG TAT GGG AAG GCGTGGATGG-3’ |
| 3’-GSPS | 5’-GAA GAG TTG CGG GCA TGGGCATACA-3’ |
| 5’-GSP | 5’-CCGTACGATGCACGCCCTTCGTTAT-3’ |
| 5’-GSPS | 5’-CGAAGCCACTTGCCTCGTCCTCATC-3’ |
| CsTLV1 gap |  |
| Forward | 5’-CACTGGTGATGTGGAACAGG-3’ |
| Reverse | 5’-AGCAAGACGCATAGCACGATA-3’ |
| CsTLV2 gap |  |
| Forward | 5’-CACTCAAAGCAGCAGCAGAG-3’ |
| Reverse | 5’-GCATCCTCGCCTCAGTGGAC-3’ |

[**Supplementary Table S2**](https://www.frontiersin.org/articles/10.3389/fmicb.2019.00626/full#SM1)**:** Virus names, acronyms and GenBank accession numbers of predicted RdRp protein sequences used to infer the phylogenetic trees.

| **Virus** | **Acronym** | **GenBank accession no.**  **RdRp** |
| --- | --- | --- |
| ***Totivirus*** | | |
| Puccinia striiformis totivirus 1 | PsTV1 | ATO91007  BAT62476  NP_620495  AAB02146  AGG68771  YP_009507833  NP_620728  YP_007697651  YP_009507835 |
| Red clover powdery mildew-associated totivirus 1 | RPaTV1 |  |
| Saccharomyces cerevisiae virus L-A | ScV-L-A |  |
| Saccharomyces cerevisiae virus L-BC | ScV-L-BC |  |
| Scheffersomyces segobiensis virus L | SsV-L |  |
| Tuber aestivum virus 1 | TaV1 |  |
| Ustilago maydis virus H1 | UmV-H1 |  |
| Xanthophyllomyces dendrorhous virus L1A | XdV-L1A |  |
| Xanthophyllomyces dendrorhous virus L1B | XdV-L1B |  |
| CsTLV1-like | | |
| Ahus virus | AHV | QGA70930  QLJ83490  APG75978  YP_009551504  YP_009333150 |
| Parry's Creek toti-like virus 1 | PCTV1 |  |
| Hubei toti-like virus 6 | HTV6 |  |
| Diatom colony associated dsRNA virus 17 genome type A | DCV17 |  |
| Beihai barnacle virus 15 | BBV15 |  |
| CsTLV2-like | | |
| Bremia lactucae associated toti-like virus 2 | BLTV2 | QIP68023  QGY72624  YP_009336942  YP_009333465  YP_009333409 |
| Plasmopara viticola lesion associated totivirus-like 5 | PvLTV5 |  |
| Hubei toti-like virus 5 | HTV5 |  |
| Beihai sesarmid crab virus 7 | BSCV7 |  |
| Beihai razor shell virus 4 | BRSV4 |  |
| ***Victorivirus*** | | |
| Aspergillus foetidus slow virus 1 | AfSV1 | YP_009508249  YP_009508251  YP_392467  YP_009508253  NP_624332  NP_898833  NP_619670  YP_122352  YP_001649206  YP_008130308  NP_047558  NP_047560  YP_004089630 |
| Beauveria bassiana victorivirus 1 | BbVV1 |  |
| Coniothyrium minitans RNA virus | CmRV |  |
| Epichloe festucae virus 1 | EfV1 |  |
| Gremmeniella abietina RNA virus L1 | GaRV-L1 |  |
| Helicobasidium mompa totivirus 1-17 | HmTV1-17 |  |
| Helminthosporium victoriae virus 190S | HvV-190S |  |
| Magnaporthe oryzae virus 1 | MoV1 |  |
| Magnaporthe oryzae virus 2 | MoV2 |  |
| Rosellinia necatrix victorivirus 1 | RnVV1 |  |
| Sphaeropsis sapinea RNA virus 1 | SsRV1 |  |
| Sphaeropsis sapinea RNA virus 2 | SsRV2 |  |
| Tolypocladium cylindrosporum virus 1 | TcV1 |  |
| ***Trichomonasvirus*** | | |
| Trichomonas vaginalis virus 1 | TvV1 | YP_009162330  NP_624323  NP_659390  YP_009507836 |
| Trichomonas vaginalis virus 2 | TvV2 |  |
| Trichomonas vaginalis virus 3 | TvV3 |  |
| Trichomonas vaginalis virus 4 | TvV4 |  |
| ***Leishmaniavirus*** | | |
| Leishmania RNA virus 1 | LRV1 | APT68186  AAB50031 |
| Leishmania RNA virus 2 | LRV2 |  |
| ***Giardiavirus*** | | |
| Giardia lamblia virus | GLV | NP_620070 |
| ***Artivirus*** | | |
| Omono River virus  Armigeres subalbatus virus  Panaeid shrimp infectious myonecrosis virus  Golden shiner totivirus  Wuhan insect virus 22  Hubei diptera virus 22 | OMRV  AsTV  IMNV  GSTV  WIV31  HDV22 | BAJ21511  ACH85916  AAT67231  YP_009256209  YP_009342432  YP_009336825 |
| ***Chrysoviridae; Chrysovirus* (Outgroup)** | | |
| Helminthosporium victoriae 145S virus | HvV-145S | YP_052858 |

**Supplementary Table S3.** Identities of the CsTLV1 and CsTLV2 RdRp amino acid sequences with similar sequences deduced by BLASTp search of GenBank.

| **Virus** | **Virus name** | **Accession number** | **Identity (%)** | **E-value** | **Query Coverage (%)** |
| --- | --- | --- | --- | --- | --- |
| CsTLV1  CsTLV2 | Beihai barnacle virus 15  Ahus virus  Parry’s Creek toti-like virus 1  Diatom colony associated dsRNA virus 17 genome A  Bremia lactucae associated toti-like virus 2  Plasmopara viticola lesion associated totivirus-like 5  Hubei toti-like virus 5  Beihai sesarmid crab virus 7  Beihai razor shell virus 4 | YP_009333150  QGA70930  QLJ83490  YP_009551504  QIP68023.1  QGY72624.2  YP_009336942  YP_009333465  YP_009333409 | 33.81  34.92  35.20  33.92  39.67  42.96  44.96  42.54  38.24 | 8e-144  9e-132  3e-124  3e-122  0.0  0.0  0.0  0.0  0.0 | 97  78  78  73  89  80  74  78  74 |

**Supplementary Figure 1.** CsTLV1 and CsTLV2 genome copy number (Log_10_ RNA/mg) in muscle, gill and hepatopancreas of the crab investigated by TEM.

**
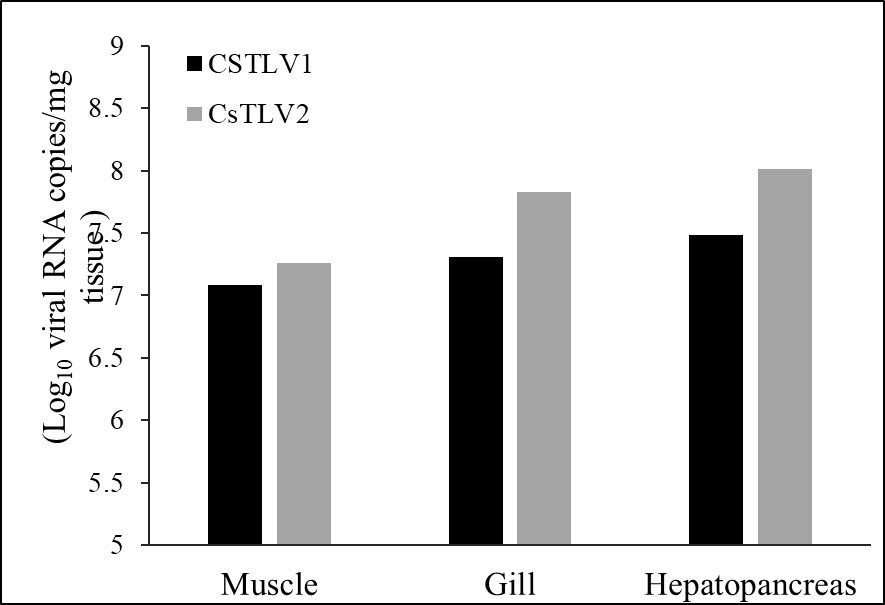
**

**Callinectes sapidus toti-like virus 1 (CsTLV1) 6444 bp**

1 TAGTGAGCGT TTGTATTGGA AGAAACAAGA GCTGGATCTC ACATCCAGTA TCCATTCAGT

61 GACTTATGGT AAGACTGAAG CGACCGTTGT AGGGCGCATC TGGCGTCCCT ATTTGAAGAA

121 ACTGCTTTGG AGGCCATCTG CTGACTACTT GTCAACACGG CTGACAATGC AGCCTGGTGC

181 AGCTGCTGCT GCATCTACCG TCATTGACCG CAACATTAGT GCTGCGGTTG GTTCTCTGAG

241 CCACTGTTTC ACCCGAGTCA TTACTCCTAC CGATCTGGGA CACCAGGCAC GACAGGAGAT

301 ACAGTCCATC GTTCACGAAG AAGTGAATAC TTGTAGATTT GAGTGGCGTC TGGCAGCCCT

361 TTATGTAATG GCAGTTCTAG CTGAAAAGCG CGGTGAAAGG ATTGCGACCC TCGGGGGTCG

421 CATGCCTGAG ATGCGATTCA TAGAAGATGC TGGTTCATTT TCCACTTTCA TGGGTGATTG

481 TCGTGGTCGT GATGTGGTGC CATATGTGTG CCGTGTCTCG CCAGATGTTG AGTCTGAGGC

541 AATATCTGTT CTTGCTGCAG CTGCTTCGCC CTTTGCTCGC GGGACATCGA CTCCGGCCAT

601 TCTGCGTAGC CAACCACGGT TGCTCAACCC AATCATCGCC ATCTTTGGGC CCATGGCTCC

661 GGTGCTTATT ACTCCGACCC TGACGTCTGG TGTGATCTGG TCAGTGGCTC AGGATTTCGC

721 GGGCCGCTAT GGTAATATTG AATTCCTCCG GGACGCAGTG AAGAATATTT GTGCGTTAAT

781 CTTCTCCCCT ACCAAAGGTG ACCCCGTCTT TGGGTCGAAA GGTATTACCC TTGCTTTGCC

841 TCCGTCCAAA ATGCATGGTC TGGCTTTGGG ACCTTGTTTG ACCAGATATC ACACCTGGGA

901 TGAGCAACCG TCTATCATTC CGCCACCAAG TGTTCAGGAG GCTATAGTGC ACGGTGCTAC

961 TGGCATGTTG TTGTTGTCCC TGGGTTACTG GCATGTGTGT AATGCTCAGT TGGTTGGTGC

1021 GGAATTGGAT GACGCTACGA TGCTCACGTT GAAGAACAGT TTCTTGCCAA CGGCGTTCAT

1081 TGGCTCCAGA GGTCAGACTC CGGTTATTGG ATGTGCTGCC CATGTTGTGA GACAGATCGT

1141 TGATGGTTTC GAGGTGGGAA AGGTCATTGG TCGACTATCG TTGATTGAGA GCAATTTCAC

1201 CAAAATAAAC AACATGTTGG TTCGGCATGC TTCCGCCCCT CAGTGGGAGG AAGTTGCTGT

1261 CTTGACAGGC ACGCTACCAC AAAGCAGTGC CCTGTTTGGG TTGATGACCC CGCTCCACAC

1321 AGCCACACCC TGCAAGGTGG GTTGTTGGTA CCGGGTGGGG GAGTTGCCAG CAGGGTCCAG

1381 CGTTGGTGGG GCGTTTGCTG GTTTCCAACA CTGTCCGGCG GTTGAATATG GTCACTTCAC

1441 GTCCGAGCCG CGTAAGATGA GAGTCAAAGT CAAACCAATT ACGTTGCCCA AGAGTTATCG

1501 CGGTGTTCAT TCTGACTGGT TTCTTGATCG TGAAATCCAG TTCCCAGAAC GGTTCATAGG

1561 TCCTGTGTTA CGGTTCAAGA CTATTGAGGG TGTTCTACAA GCCCATGCGG GGAAAACATT

1621 CACTCATCCC GTCAAATGGT ACTTAGAGAA TTTCGTTCCA GCAGACTGTT TGATGCCTGG

1681 TGTGGTGGGT TCAGCTCACA GTCTGGAGAT TGTCGAGGAC GATGAGAATG ACAGTGATAG

1741 CGATTGGGGG GCAGGTCCGT CTGCACCTCA GCGTGATGAT GGTGGTGATG ATGATAAAGA

1801 TGATGATTCT AGCAGCGGTG AAGACACGGA TTCTAGGCCT CAACGGGCTG TTCTGGCACA

1861 GGAAGTGGCA CATGAGGGTT CGAATGATGC TGTGGTTCAT GGAGTTGATA AGGGTGAAGC

1921 TATTGAAGTG GTCAAACCCA AAGAGGCATA CGAAAAAGAG GCTGAGGCAG AAGACGACAC

1981 TGAGAGAAAG AATAAACTTC TTGAAGATGC TGGGTCTGCC CCAAAAAGTG GTGAGTTTGC

2041 TGATGGGCGT TGGATTGGTG TTCGCGGTCG GAATCATGCT CGCCGTCTCA AGAATGCTGA

2101 GAGGAAGGGG CAAAAGGTAG TAGGCAAACG TGTTCGGAAG GTTGGTGGTG ACGTTGGTAC

2161 ATTCACCGTG ACTGCTGACA CCAAATACAA GAGTTTGGCT GCCGCTGCTA TGGCTGGTAA

2221 GGTCACACTG GATGATCTGA AAAGCATCCG GTCACTACCT GGTGGTAAGA GGTTTGTTAC

2281 CGCAATGCTC AGTGGACCTG CAGGGATCAC TGGTGATGTG GAACAGGCAT GCGAAGCGGC

2341 TTTGAAGCTT GCTCTATTAG ATCGTGATCA GGGTAGTGAA TGTACTAGCA AACGTATTAT

2401 GCTCTCTGAT GCTGCAGTGA AGACCCTAAC TACCAAGGAA CCTCTGGATA TGCTCAAGAA

2461 GATACCCATC CGAGAGAGGA AATCAGCATG TGATGTGCTT CACAATGTGT GTAAGTTCGT

2521 TCAGCTGAAT GTAGACTCAC CATCTGGACG CCTCCAGCTG GAGAAAGATG CTTGCAACTT

2581 CAAACACTAC GCTTTTGGTT TCCAACAGTG TGGTGCCCTT GATCTTAAGG AATTGTGTGA

2641 ACACGGTGGT TTTGATCCTG ATAAAGTAGG CCAATTGGTG ACTGAAAGCG ACATTAGGAG

2701 GATGGTTGCT GCTGGCATTG ACCCCGTCCG TGAGGTCAAA TGTGCTATGT CTGGCGTGCG

2761 GACTGCTCAA GATCGTGGTA AGGACACTGG TGCTGCCATC GATGGTATGA AGGGGCGTGT

2821 TCAAGCCGAC ATCGAAGCGC AACATGAGGC AGCCTATGAC GAACAGACAG CGGCTGGAGT

2881 GGAGATGGTT GTGGCAGGTC TCTGTCCACC TGAGGTGGCA TGTGAAGCAT TTGGGCTGAC

2941 TATGGAAGAA TTAGCTGAGA AGGCAGGGAT GACTATTGCA CAGTTGAAAT CGATGGGTGG

3001 CGATGCCGGT GAGGAAGAGG TGAATGGTAA CGTCGGTGAG ACAGAACAGA TGAAGATGGA

3061 GCCGGATGTG ATCCATCCTA CAGAATTGGC CGATTCGGTG GTCATAACAA GTGAGACCCC

3121 GGGTGCTAGT GAAGGTGATG TGGGCAAGTC GGTCCACATT CCCGCCCAGG CTGCAGCAGC

3181 AGACTGTGTC CGTGAGAAGG ATTTTGCGAC GGCTTCGGGG AGTACTTCCG GCTTGCCTGC

3241 TCAGCAGTCG GACAGTGGGG ACTCTTCGGA GCTGGAGCCG GGGGTGGAAG TGTACACGGA

3301 ACTTCCACCA GCAATGGACA TGGTGATTGC AACCAGCGCA TCTGGGATGG GAGCCGCACC

3361 AGGTGTGAAA TTGGCAGAAA AAGCAAAGGA GTGAAGGAGT GGGATGTATG CGGTAAGAAG

3421 AAGCAAAGGA TGGTTGATGT ACTGAAGGAA ATTGAAAGTT GGGAGGCAGA GATGTGTGTT

3481 TTAGGGGGGG GGATCCCTTT GGGGTCCCAC GCCCTTAAAA CCTTTTTTTG TTGCTCAATT

3541 GCGAGTGCTG AAGACTATCG TGCTATGCGT CTTGCTGTAC GGCTTCCCAC TGGGCCTGGC

3601 AATGTAAAGA GCATGCGTGG TGTGGTCTCA CCAGAAAGTA TGAAAGAATG GTTCGGCGAG

3661 AGCTTGGTTC CCAAAGGTAG CATTGCATTA ACCCTAACTA CAGTGTACAC GGTGTTGCGC

3721 AGGGACCCCA CTCTCTGGGA ACGGGAACTG ATTGGCGCCA ACCGGAATGG GCAACAATGG

3781 TCTGCGGCTG CTATGATATT GGGGTTAAAC TGTTTGCGAG CACCCCTTCT GGCGGCAATC

3841 GTCCGTGCTG GCTGGCAACG TATTCCTCTA CCCGACTGGA CAACAGTGCT TAGTGACTGT

3901 GTAGTCGCAT TGAGACGTTG TCCTTTTGTG GAGGGGTGTG GTTGGGAGGA ACCTGACGGT

3961 TACTCATTGC GCAAGCTACT CAGTTGCACT AATCGTACGC TCGATGAGGC TGACTGGAGT

4021 AAGGAGTTGA CGAATATTCA ACGCGATTTG CCCGTCCACT ATTGGGTTGA CGAATACGGC

4081 TGTAGGTCAC GTGCGCTATG GCAACCCAAG ATAGTCAAAA TCATGCGGGA AGTACTTGAA

4141 CGAGTAGTGA ACCAAACACT CAATGGTCCA CCACTAGAGT CGATGGAATC ATGGTGGTCG

4201 AGCCGGTGGG CTTGGGCTCC TGGTGGGTCC ACATCCCAGA GACATGTATT GAAGGATGCC

4261 ATCAGTGCTG ATGAACGCTT GACTCAGGAT GATCGAGCAG GCAAGAAAGC TGTGTTTGAA

4321 AACCTTGATG ATGATTGGGT ACAGCGGGTG CTATGTGACC CTCCTGCTGT TGTCGGTCGT

4381 GCGTCTACTA AACATGAGCC TGGTGCTAAG AACCGAGCAT TGTATGCCGA AGGCGATGAG

4441 AGTTTCTGTG TTTCTGCGTA TGCATCGCTT AATTTTGAGA AATTTATGAA CGTTGGCGGC

4501 ATGGTTGGCA AACAAACACC TGCAGATGTG TGCGACTGGT TATATGCGTC AAACATGTCT

4561 AAGCTCAGTA ATCCGTATTG GTTGTCTGCT GACTACAGTG ATTTCAACAA GGGACACGAG

4621 GCGGAAGACA TGTGGCTGCT GGACATGATG GTTGCGCGTG CATGGGCTGC AACCAATTCC

4681 AATCCGCTTG TCCGCAGGGA CAAGGTGCGT TGTGCAAGAT GGACAGCTGG GAGTCATCAG

4741 GTCAAGTGGG CCAGGATTGA GGGCAGGATG GAGCGTGTGT TCTCGGGACT GTTCTCCGGC

4801 AGCAGGAACA CGGCTAGGGA CAACACCATT TTACACAAAG TCTACTCCGA GGCGATTCTC

4861 TGTGCTATGC GTGATGCTGG TTGGAAGGGC GAAGTTGGCT ATCAGGCTTA CTGTGGTGAT

4921 GATGAAGACA TGGAGTTCGA TAATTGGCGT AGTGTGTTAT ACTATTACGT GATGCACTCT

4981 TTGAGTGGAC ATGAGCTCAA ACCACCCAAG CAACTGGCAG GTAAAACGCA CGAGTTCTTG

5041 CAGCGTATGG TCATTCCGGG AGAACACACA ATCCGGCCGT TGTTTGCTAT GTTGGCACAG

5101 GCGGCTAGTG GTAACTGGTA CAATGATACG TATACGTGGT ATGGCAATTC AATCCCTGCG

5161 GTCAGAGACA TGTGTTGGGA GATGCATGTG CGTGGCATGC CCCTCGAATG GGCGAGACGT

5221 TTGTGTATCG AGACTTTAAA TGCTACAATG CGAGTTCCAT TGGGTAACAA GGAGTGGCGG

5281 AAGTTAGAAT GGTGGAGCTA TCGGAACGGT GGTGATGTCG TACACCCAAT GTGGAATGGG

5341 TTGGGTATAG ATTCTGGTAC TCAACCACGC CTGACGCCCA AGACGGTGCC TGTCCAAGAG

5401 GTGCCACAGA AGGCAAGTAA TGCATGGGTT GCTAAGGTCG AGAAGCTGAC AGGCAAAATT

5461 GGTACGGATC GGAGATTGGA GATTATTCGT GAATGCAGTG CTGACAGTTA TAGCAAGCTC

5521 TATGTACACA CTCGTTTGGT GAAACAGAGG GAGGAGGTGA TGGAGATGTG GCCGGAAAGG

5581 ACTAGTGAAA TACCCGTTGG AGCCCTACAT GTGGGTCTAA TCACTGCTCC ACCCCTACGA

5641 CGCATATTGC AAGGCATAGT AGCTGAGCCG ACTGATCGAC GCCCCGTCTC ATCTGATGCA

5701 GTTGCTGCGC GTTTCGGTGT CAACGAGAAG ATTGTGTTGG CGTGTGGCGG ATGGCAGGAG

5761 TTCTTGCGGT TGGTTCCCCC TGAGATGGTA GGCAACTACG AGGAGCCTGT TACCTCATAC

5821 ACTATACCCC TATGTTACTA TAAGATGGAC TCAGCATTAG TGTCGTGGGC CCGCGTCAAT

5881 CGGTGTCTGC AATATGAGCC AGACCGGCGT GCTGAAGCTA ATCGTGTCTG GGCAGAGACG

5941 GTTGGGCGAG GCATCAAGGG TAGGCATATA ATATGGTGGT TGGTCCCCAA TGCTGGTGGT

6001 AAAACTACGT ATTCGCGGAA ACAGAATCTA ATCGGTCGAC AGTGTATTGA TATTGATGAT

6061 TTATTGATCG AGTATCCTGG TATGCGTAAC ACGTTGCGTA GTGTCCGGAA GGGTCATGGA

6121 GATGGTGGAC AGAAAGACAT CCTCGCTGAT TTGATTGCTG GTGTCGTACA AAAGAATAGT

6181 GCCGACGAAG TGTTGTGGCA AGGTGATGCA AGCTGGGTGA TCGGGCGGTT GAGGAAGCAT

6241 GGTTGGGATG TGGATCTGCG TATCGCGGAT ATCGATGAGG AGGAGGTGGT TAAACGTTTG

6301 AGGTGTCGGT GGTGGCGCGA TGAGCGTATT GAAAAAAGAA GGTTGGAATG GTTGGTTAAT

6361 GTAAATAAAG CTCTTAGTCA AGGTGCAGTA GTATGCCCTT TGGCGTGATT GAGCGGCAAG

6421 GTGCAGTTTT GCTGCACACC CCCC

//

**Callinectes sapidus toti-like virus 2 (CsTLV2) 7421 bp**

1 GAACGTCAGA CTGCGTTGTA AAATAAGTGT CTAAAAACCA TGATGGCTTC AGGTGAGCCT

61 TTGCAATCTC AAATTGTTGA GCTTCAAAAC GAACAAATTG TAATGAATGA AAACACCCAA

121 GCGAACGAGG TCGAGCAAAC GGAACAGAAC GTTCCAGCTC AAGGGCCTCA CACAATTGAA

181 CAGGTGGTCG GACACCCAGC TGTTTCTCTT ACTCCGAGTG TCCAACAAGG AACATATGGT

241 ACCATGCCTG ATCCACCCGG ATTGCCCATG GGCACCAGTA ATGAAGTACC AGCCGGAATT

301 CAACACGCGT TGCTTGACGG CATAGGAGAT GATGAGGACG AGGCAAGTGG CTTCGTGTCT

361 GGGAGAGAAT GGAAAGCTAG AGTTCCGTCC ATTGCAGGTG GGCAGGAGCA GCTCAAGACC

421 ACATTCGTCA AGGTTGGCAA CCTCAACACA GCGATGGAGT ATCCAACGGA AAATCCATAT

481 TGCTCGATGG ATGTGGAGGA TTTCAGGAGA TGTGAGGATG GAGTGGATAT TGCGTTGAAA

541 CCACAGTCCT TTAAGGATAA TAGGGTCATC GTCGGGATAA CGAAGGGCGT GCATCGTACG

601 GCCCGTCAGT GTAAATTGGA CTATGTTCGA TATGATGGGG TCATGACGGT AACTCCTTCC

661 TTGTGGAGGT CACAGAGAGT CATGCTGACT CCGGAACCTG GTTATGCAGC AGTGTTCCTC

721 AGCGCTCTCG ACTCGGATAA TTTGGCTGCC AGGCGTGCTC TCAACCAGGT GTTTAATGAT

781 GCCTCGAACG TCTTGGCGGA TATCGGAGCG GCTGCGGCAC ACATGCGCGA GATGTTTGAG

841 CGGCCGGTGG AAGTTACGAG ATACACGATG AGGTTGGCGA TGATGTATGT AGAAGCGAGG

901 TTGTTGAGCC CAGACCCTCA CGGAATGGTG ACCTTCGAGT CGCCTGATCA GCCTGAGTGG

961 GTCCGAGCGA CTAGCTGGGC GGCTTTCAGA AGGGAGGTGA TCACAGATCA GAAGCGTTTC

1021 TCGGAGGTTA ATTACATTCC TTGGATGTCT ATGACGGCTT GTCAATTGCC AATGGAGGGA

1081 AAGCTGGTGG AGGTGCTTGC TGCTGCCAGT CTGCCCAACC TTTCGTTGAA AATTGGTGAC

1141 AACTACAAAC CGCTCATGTG CTGGCCTTCA ATCAACAACA CGAGAATGAT TACTAACACT

1201 GGGTTCTTGG CTTCGACGGA ACAGGGATTT TGGGAATTCT ACCCTTCGCA AATATACGCC

1261 GCTGCTCGTG CATGGTGCGG TAGGTACGGT GATGTTGATC TTCTCGATCA GCTTCTCCAG

1321 TTCTGCTTCA TCATGTACTG GGGCGACGCC CGTCGCGGTG GTATCAATGT TGCTTCGGGG

1381 ATGACTGACT TGGCCATCTG TTTGCCGGTA GCTAACATGC GGGGGTACTT GGCTGCGCCT

1441 TACTTGGCAG TGCGTGATGT TACGAGAGAT GTCTTGCGTG AGATAGGGAC GGAGGATCAT

1501 AAGAGGCAGG TGTCGGAAAG CGTGTCTACG GCGACTTTGT TGAGTTTAGG GCATAGTTGG

1561 GTTAGTTGGA AATACACCGA TTATCTCATG CTTGCGGAGA AACTGAACGA GACAGAGATC

1621 GAGCGGAGAC TCGGGATGGT CAGGATCCAG AGTGATCGAG TGCCAAGGTG GGGAATGGTG

1681 CAGCAATTCC TCAATGGTCT GGGTTACCGT GGAAAGGTCG GGAATTGGTT GTCAAGAGTT

1741 GGATCGATGG TCACAGTGCA GAAGTATACG CGTGGATATA TCGAGCAGAA AAGAGTTATG

1801 ACTGGTGATG TCATTCCGTT CTTACCTAGG TTGCCCGATG GTTGCGGAGC ACAAGGATGG

1861 ATCAGTCCAA TGCCGGTGCC TGGGGATCTC GGACTTGCTA ATCAAGCGGT GGTATTCTCT

1921 GATTTGCCAA AAGCGGACAT GAGGAGGATG GTCGCCTCGC TGGATGAGCT GAGGAACATT

1981 GATTTCTGGT TCACAAGGCA ATCGCGCACC TCCTTGACCT TGACTCAAAC TGCTGCTCCA

2041 CGACCCAAAA GAGCGATGAG TGGGGTTGCA CCTGATATGC CCTTCATGGC CGTGACCGTT

2101 CGGGGGGGGG ACCGGATTAG ATGGGGCTTC ACCATCCCAC AAGCTTCGAT AGGACCAGCA

2161 ATTTACGGGA CGCACGATCG CTACAATCTC GATTGGTACG TGATGCGTGG GAGTGCGGAG

2221 CAGAGAGGGG TGTTCATCGA GACGCCGCTT GAAACTGAAC CGGTCAATAT TGGAAAGCAG

2281 GAACAATCTG AGGATGACAA GTTAATGCGT GCGCTCGAGG ATCAACGTCG AACGCAAGAG

2341 CACAAGCACA AGATGGATCT GGCAAGGTTG GAGAATCAAC TTAAGATGGA GATGGAGAAG

2401 CAGCAAGCCG CACTCAAAGC AGCAGCAGAG AAGGCAGCAG CTGAGGAAGC GACCCCCTCG

2461 CCCCCACCCC CCCCGAAAGC AGCGGGACCT CCACCTCCCC CGCCTGCCCC AAGCATGAAG

2521 ACCCTGGGCA TATCTCAGAT GGAGAAGCCT TCGGGTGGTG ATTTCTCTGA GGAACTAAAG

2581 GACTTCGCTA CAATGGGGAG GCGTGAAGTC GATCAGATGG AGGAAATGTT GCAATCGCAG

2641 CGTGACGCAG TGATGGAAGA GGCGCGGCAA AGAGATGTTG AACGAAGAAA AGCTGGATGG

2701 TTCGGAAAGG TCGAAGGGAA GAAGAAGGAT ACTTCCGGTA CGTGCAGGGG GCCATGGACG

2761 GAATGACTAA ATGGCGTGAT GATGACAAAG ACGGGCCTGG GGGTGGAGGA AAGTCTAAAA

2821 GACGCCAGAT GGACGCCCCA TTGACGGCGA CTCGGACGCA AGCCACGGAT GGGGGGAAAC

2881 CAGCATCCAA AGGTGGACCT TCAGGAAAGA CGAGCAAACG GGGAGAATGG CCAACACCAC

2941 AGCAACAGAG AGCAGGTACT GTCTTCGCCA TCAGCAAGAG AGTGCGGGGC AGGAGGCGAG

3001 GTAACGAGGA TGATGTAGTT ATGATGGGTC TCGGTTTGTT GCGGTGGAGC ACAGGACTGG

3061 CGGTGCAGAA AAAGCAGTTG CAGGTGTGTG AGACTCCAAA AAGGCCGACT TTCTCTGCTG

3121 ATGATCTGGC TGCTTTTAAG AAGCGAGCAT TGATTGAACT GTTGCGTGGC AAGGAGGAGC

3181 TGGCAGCGGC GGCACTACGT GTGATGTATG ACCATGATAT GCGATTTGAT CACGAGAGGG

3241 TGTTGCTGCG TGTGTTGGAT AGATGTCTCA TGACGGATGA ATCGATTGAT GAGGAGCAGT

3301 TGGATTTGGA CTTCTCAAAG AACGGTGAGG ACCCGGGGGC GCCTGACGCG GCAAGGCTGA

3361 AGCAGTTGAA GGAACAAGGA ATGGAATCGA TGAAAATGTT TGGTTGGCAT CAGAGGAAAT

3421 ATGATGATGT GCAACAATTG TACATGAAGG CGTTGCGCCG AATGGTACGT GCAATGCCGC

3481 ATACTACGAT CGCATCATTG GAGTCAATGT TGTATACGGC TTGGGTGAGT GAGCAGGGAC

3541 AAAGGATATT GCGTGGTAGC GGATTGATTT TCGGAGGTGG TTCAGAAGTG ATGTCTACTG

3601 AGGCGAGGAT GCTGATGATG ATCGAGGTTG GAGCGTACAA GGGCGAACTG TGGGAGCCTC

3661 GCTTGCAACA ATTGCTCGGT GAGACTTTCA ATAAAGGAGT TATGGCTGAC TGTGTCCATG

3721 TGGAGGATGG CCTCGCGATC GATTCTTGCC ACTGTAGTGG TGTTAACTTC GGTGGCTTGG

3781 AACTTCATGA CTGGATGAAT CTGGATCAAA AAGGTTTCTT CACACGACGA TCGAAATATG

3841 AGCGGCAACG CATTCTGATG TATGGTGGTT GTCCGAAGAG CCTGGTCCGA CGTTACGGTG

3901 GACGATGGGC TGGGAGACGA AGAGATTTGG ATTCACGGAG GGCCAGGAAG ATGGCTGTGG

3961 AAGATATGGA CTGTACACAG TGGGAGCTGA CGGGGCACAA GATCTACAAG TGGTTTGAGA

4021 GCCTCAGCGA ACGCAGACGC GCAGCCTACT TGGCGGAGTG TCATCCGGGT TCGCTGGGAC

4081 CAGAGCACTT TGCCGAACTG AATGTGTCTG TCTTGTTCCA GCAGGATGAT GAGCAAGCTA

4141 TGGCGACTAC ACGGCGCAGG AAGGAAAGCT TCGATGAATG GATGTCTGAG AAAGATAGGT

4201 CTGGGGACCA GCTGGCCGAG AATGTGTGTG AATGGATTGC AAAAGAGTGG GGCAGTAAAA

4261 CAGTCCGCGC CTCGGAAATG ATACCATCCC CCTTAGTAGG TGGGATTCCA ATTGTCATGA

4321 CGGCGGCCAT CCTGTTGGAA GCTGTGCCTG CAGCGGCTCC CCTGATCGAG TTGTTGCATA

4381 ATACGAGCGG TCAAAGTGAA GAAGGAGTTG TTTGCGGGAC TGCCTTCGCG GTGTTGTCAC

4441 TCCCGTATCC CGTGTTGCGT GAATTCGTGT GTAGCACTTT GTTTGATGAG CCTTTGCATG

4501 AGTGGTGGAC CACGGCAAAA CAGCATTTCG TGAATGTGCG CAGGACTTTG ACCTGGGGCG

4561 ATGTGCGTAT GGATGAGAGG TTATTGATGC TGCGGAAGAT ATTAAATGTG ACGATTAGGG

4621 TCCGAGGAGA TAGCGACATT GATAGTGAAA ACTTCAACCG AGGGTGTGTC GTAACGCGAA

4681 AACACGTGTG GGGTCACTCT GTTCCAGAAG ACGGGGTGAG CGCTTACTCC TACTACGAGG

4741 ACAAATTTAC GAAGTGGGCC GGGACTGCGG TAGCGGAATG TTTTGGGAAG TACAACAAGG

4801 AGATACTGAG TCGAAGTCCA GACGATTTCT GGTGTGAAAG AGCAGTCCGG GGAGCTAACG

4861 GTGCAAGCAA GAGGATCAAA AACGTGGAAT GGAACGATGA GAGGCTTGGA AACCAGGATC

4921 GACCCGGGAA GAAGGCATTG GTAGAGTTCT TGGATGACGG GGCTTTGAAC AGAGCCATGG

4981 ACGGCACGCC TTGCAATAGG GCCTATTACT TTGTTAAACC TGAGCCCGGA CGCAAATTGC

5041 GGTCACTGTA TGCGACTTAT GATGAGGAGA GTTTTGTTGC AGCGTATGTC AGTCAAGGAA

5101 TTGAAAATTA TATGGGCGCT AGCAAGGGAG TCATGGTGAG GCAAACACCG GCGGATGTGA

5161 TTGAATGGAT GGCAGTGTCA AAGAGCGGGG CGTTGAGCGC GGAAGCAAAA GATGCGTATT

5221 GGTTGTCAAC GGACTATTCA GATTATAACT CGGAACACAC AATGTTCGAA ATGCAGACGC

5281 TGGACGCACA GGTGGCAACC AAGTTGGAGG AAGCTCCCCA ATCATGGAGG AATGTGCCTA

5341 AGGCGGCTGC GGCTTGGTGG TTGGTGGCTG CGAAAAGTAG GAGTTGTATC ATGTACGGAA

5401 AGGGCAAGAA GTTCGAGGAT CATGATGGAT TGTGTGAGAC GGAGAAGCAG GGGCGTTGGT

5461 TGCGGTCAGT CAATGGTCTG TATTCCGGGT CTCGCAACAC GGCTCGCGAT AATACGTGGA

5521 TCCATAGGGT ATCAGTGAGC ATTGCGATGC ATGAGAGCCC GGAGTTTATG GACAGGAATG

5581 ATTTCAAGTG GTTCGCGTTG TGTGGTGATG ATGAGGATGT TGCTTTTAGG GATGAATTGG

5641 CGGCAGCGGG ATACTATATG ACACTCGGAG CGGTCGGACA TCAGCTCAAC CCGGTGAAAC

5701 AGCTTGCGGG GCATAAGAAT CATGAGTTCC TGCAGCTCAC GGCAACCAGA GAGGCTCGGG

5761 TTGAGAAACC ATTGTGCAGC TTGCTGGCGA CACTAGCGAC CGGGAACTGG TACACTCAGC

5821 TCGGAACTTG GATTCAGACT GCACCAGTTG GAGTAATTGC CAATTACTGG GAGCTGTTTT

5881 GTAGAGGTGC CAAACTGCAA CATTGTCGGC GTCTAGCGAG AGCAACACTA GATAGGCTAA

5941 TGCGTGTCAA GAAGGTGAAT GAAGACGGTG AGACTGAGGA AAAACTCCTG GAATGGTGGG

6001 ACTACAGATT TTCTGTCGAG GTGGCTCCCC TCTTCACAAT GCATGAGGAG GATGAACCCA

6061 AGAATTTACC GCGTTTCGAG GCGGTTGCAG AGGTTGATAA AAGCTGGGAC AACAAAGCAT

6121 CGAAAGCATA TGTCAAGAAA CACCGGGCTA TGTTGAAGCA TCTGCCCAAA CGTATCGAAA

6181 GTGAGTTTGT GGAGAGCGTG CAGGGACAGA CACTTGGGGC TTGTTTGAAG ACGTGGCAGC

6241 AGAAGGAAGC TAAAAGGTGG TGTGCGCGAC ACTGGCCGGA GCGAGAGAAC GAATCGATTG

6301 TCGAGGTTGA GAAACGAGCG GAATGGAAAA CTGCTGTGCC GGATGAAGTG CGCTTGGACG

6361 AAGTAAGGCA TGGGTATTCG GGTAAGAAAG AAATGATGAC GGAGGAAGCA GTCTGTGGAA

6421 GAATGGGAGT ACCGTTTTTC GTAGCAAAGA GGCTCGGCGG AATTGAGAAG CTAGGAGGCA

6481 AGGTGAAGCT GGAGCAATGG GCTAAAGCAT GCAACCTCAA CGCCAAATTC GTTCCGATCA

6541 ACGGAAATGC ACACAAATTG CAGATGAACA TCAGGGCAGC GATGACATGG GCTAAAACTC

6601 CGACAACGCG GTATCATGGA GACAAGCGAC ACTTCACTCC GAAGAAGCTG TATTATGCAT

6661 ACGTCGGGAA TGGAGGAGGT AAATCACATT TGGTGCGGAC GACAAGAGTG GCGAATGATC

6721 TGGATGAAGT GTGGATGAGT GAATTTGGTT CAATCAGGGA GGCGCAAGAA GCACAGGCGC

6781 AGCGAAGCAG TTTTGGGAGA GTGACCAAGA CGATGCAGCA ATTGTGTGAA AAGGCGTTGA

6841 GCAGAAACGG GGTGCTGGTT GGACAAGTGG ACCCGAGAGA GATCAAGAAA GCCTGCGAAG

6901 TGATCGGGAT TGAGATTGAA ATTGTAACTT ATGACCCAGG AGCCGAGGTA CGAAGGGAAC

6961 GTATGGGAAG GCGTGGATGG GCAGAAGACA AAATAGAGCG GCGAATGGTG AGATGTGACA

7021 AAGTGTATAG AGAGGTGGCC GATGACCCAC AAATTAAGAA GATGCATGAC TATGAAGAGT

7081 TGCGGGCATG GGCATACAAA AGTGACTCAC AGGCCAAGAT GGAAATGCAC AGAGGGGTTG

7141 GAACCACGGA GAAGATCCTG CCCAAATTTG TGTTTGACCG GACTGAAGCG GTGACGAGAA

7201 AGATCGAGCT GGAGGCCGAG GACAGAGAGG AGCAAGGAGA GGAGAGAGTC GAGAAGAAAA

7261 GGAGTCTGAT CAAAGAGAGG CAGCGCATGC GGAATAGACT GCGGCGCAAA AGAAGAAATC

7321 ATGAAGCAAG GATGGAGTGA TGGATGGAAG TATTGGTGTT AATGGTATTT ATTTATAAAA

7381 CAAGAACAAG CAGTGATGCT TCCGACCGAG CGCGGAAGTT T

//
